# Steady State Free Precession NMR without Fourier Transform: Redefining the Capabilities of ^19^F NMR as a Discovery Tool

**DOI:** 10.1002/anie.202422971

**Published:** 2025-02-10

**Authors:** Jeremy R. Gauthier, Flavio Kock, Katelyn Downey, Tiago Moraes, Luísa Souza Almeida, Derek C. G. Muir, Robert J. Letcher, Luiz Colnago, Krish Krishnamurthy, Scott Mabury, Andre J. Simpson

**Affiliations:** ^1^ Department of Chemistry University of Toronto 80 St George Street Toronto, ON M5S 3H6 Canada; ^2^ Physical and Environmental Sciences University of Toronto Scarborough 1265 Military Trail Toronto, ON M1C 1A4 Canada; ^3^ Departamento de Ciencias, Química Centro de Espectroscopia de Resonancia Magnética Nuclear (CERMN) Pontificia Universidad Católica del Perú Av. Universitaria 1801 Lima 32 Peru; ^4^ Department of Biosystems Engineering São Paulo University 11 Av. Páduas Dias Piracicaba, SP 13418-900 Brazil; ^5^ São Carlos Chemistry Institute São Paulo University 400 Av Trabalhador São-Carlense São Carlos, SP 13566-590 Brazil; ^6^ Canada Centre for Inland Waters Environment and Climate Change Canada 867 Lakeshore Rd Burlington, ON L7S 1A1 Canada; ^7^ National Wildlife Research Centre Environment and Climate Change Canada Carleton University 1125 Colonel By Drive (Raven Road) Ottawa, ON K1A 0H3 Canada; ^8^ Embrapa Instrumentation 1452 Rua XV de Novembro São Carlos, SP 13560-970 Brazil; ^9^ Krish Krishnamurthy, Chempacker LLC 3054 Beckley drive CA 95135 USA

**Keywords:** CRAFT, fluorine, NMR spectroscopy, sensitivity enhancement, SSFP

## Abstract

The 2024 Zurich perfluorinated compounds (PFCs) summit reiterated the urgent need for non‐selective analytical approaches for PFC detection. ^19^F NMR holds great potential, however, sensitivity limitations lead to long analysis times and/or the possibility of not detecting low concentration species. Steady State Free Precession (SSFP) NMR collects the signal in a steady state regime, allowing 100’s of acquisitions in the timespan of a single traditional NMR scan. Unfortunately, data truncation from SSFP leads to artifacts and spectral broadening with Fourier transform, hindering interpretation. When non‐Fourier based time‐domain analysis is used, namely, complete reduction to amplitude frequency tables (CRAFT), limitations of SSFP are eliminated while sensitivity gains are retained. This work introduces the combined approach, then applies it for the measurement of PFCs in environmental and biological samples. In all cases, the approach reduces analysis time from many hours to minutes and/or greatly increases the range of compounds detected. For example, when PFOA was spiked into human blood, the detection limit improved ~50‐fold vs standard NMR, while in a standard mixture, the approach detected compounds missed by LC‐MS/MS. The technique can be adapted to any nucleus providing a facile approach to reduce experiment time and improve sensitivity of NMR in general.

## Graphical abstract

Steady state free precession (SSFP) – Complete Reduction to Amplitude Frequency Tables (CRAFT) not only improves sensitivity, resolution and number of signals detected by NMR but can also dramatically reduce experiment times. This powerful approach unlocks the potential of NMR as a discovery tool, enabling unprecedented detection of fluorinated compounds in environmental and biological samples.

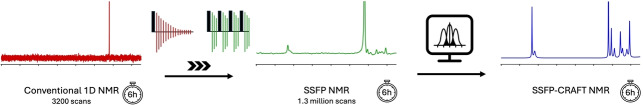

## Introduction

Nuclear magnetic resonance (NMR) spectroscopy is an essential non‐selective detector with the ability to solve novel structures from scratch without the use of libraries. If for example, environmental research is restricted to libraries of previously identified molecules, then, as a field, we are always looking for “what we already know” and potential transformation products, or new classes of pollutants, can go undetected for decades. For example, in 1976, Prof. Donald Taves performed ^19^F NMR of human blood and found “*widespread contamination of human tissues with organic fluorocompounds derived from commercial products*”.[^1^, ^2^] But as NMR is underused for environmental discovery, it was not until decades later that these PFCs were ‘rediscovered’ using mass spectrometry (MS) approaches; they have now reached ~50 ppb in the general population and as of 2013–2014 up to ~4000 ppb in the liver of Hudson Bay polar bears,^[3]^ which is far above the levels that are suspected to induce health issues in humans.[^4^, ^5^]

Currently, the number of unique per‐ and poly‐fluorinated alkyl substances (PFAS) is estimated to have surpassed 7 million compounds.[^6^, ^7^, ^8^] Of notable interest is the growing discrepancy between targeted measurements of PFAS and total fluorine measurements. This discrepancy in the known mass balance of fluorine has been observed for more than 20 years, beginning as early as 2001 when ^19^F NMR spectroscopy revealed nearly 10 times the concentration of PFAS in an aqueous film forming foam (AFFF) contaminated river than tandem mass spectrometry.^[9]^ Similarly, this discrepancy in the known fluorine mass balance has remained over subsequent years, even as mass spectrometry methods improved.[^10^, ^11^, ^12^] ^19^F NMR spectroscopy has been recently demonstrated to help close this unknown fluorine mass balance. As ^19^F NMR has less bias towards the types of functional group chemistry, matrix, and sample type, it has been able to reveal additional sample detail.

Despite the advantages of ^19^F NMR for analysis of PFAS in the environment, it is still limited by low detectability of signals above the uncertainty in the observed data, more commonly referred to as low signal to noise ratio (SNR) (height of the signal / height of noise), or having a high limit of detection. There are several strategies for improving detectability of a signal in NMR including the use of higher field strength magnets, cryogenically cooled probes, or noise reduction processing.^[13]^ However, these approaches either are costly to purchase and maintain, or are non‐trivial to implement.

Steady state free precession (SSFP) NMR is a different approach which allows for the acquisition of thousands of scans in a short experiment time in any type of spectrometer.[^14^, ^15^, ^16^] In this approach, spins are subject to a fast train of radiofrequency (RF) pulses spaced by a time between pulses (T_p_) much shorter than the transverse relaxation time (T_2_). The exceptionally fast acquisition results in samples whose magnitude never decays to zero, and the most intense section of the FID is continuously sampled. The spins remain in a steady state regime throughout the entire acquisition, permitting a large number of scans and an increase in NMR sensitivity.[^15^, ^17^]

Despite its capabilities, SSFP also has limitations that must be addressed. The severely truncated FID collected during the SSFP experiment leads to serious spectral truncation with Fourier transform, resulting in spectral artifacts and increased linewidth. As a result, SSFP has predominantly found use in imaging, where such limitations are of little consequence. SSFP has only been applied sparingly in high field NMR spectroscopy.[^15^, ^16^, ^18^, ^19^] Interestingly, all these limitations can be circumnavigated if Fourier transform is avoided.

NMR data are collected as time‐domain signals (free induction decay; FID) and are arithmetic sums of several decaying sinusoids, each one described with four unique and fundamental NMR parameters (frequency, amplitude, decay rate and phase). These four parameters, for all practical purposes, of each sinusoid are orthogonal to one another. FIDs are usually Fourier‐transformed (FT) into frequency‐domain spectra which enables a clear visual analysis. The time domain data and its FT counterpart have the same information content. The spectrum is a representation of the same four NMR parameters in a complex representation, defined by a Lorentzian equation. This mapping of intensity as a function of frequency (i.e., a two‐axes representation) requires that the phase be adjusted to remove the dispersive components before frequency, amplitude, and decay rate be measured properly. Fourier transform of severely truncated FIDs, such as the SSFP data, results in significant sync wiggles, increasing the resultant observable noise in the spectrum. In addition, the signal linewidth is increased (over and above as dictated by the natural decay rate), resulting in decreased height of the observed signal in the spectrum. Thus, the detectability of minor signals is compromised two‐fold by the very nature of the process (i.e., FT).

CRAFT (Complete Reduction to Amplitude Frequency Table) is a data processing method that exploits the orthogonality of the NMR parameters in time‐domain and extracts all signal characteristics directly from the FID. CRAFT utilizes a Bayesian modeling process to decimate the complex time‐domain data into a table of frequency/amplitude/decay‐rate/phase for each sinusoidal component in the FID.^[20]^ The CRAFT table thus represents the FID in a tabular format and can be used for data interpretation including but not limited to visualization (i.e., spectrum). A recent review presents several 1D and 2D applications of CRAFT and the associated benefit and proposes a new paradigm in analyzing NMR data.^[21]^ In this approach, for example, the detectability of a signal stems from estimating an exponentially decaying sinusoid, defined by the four orthogonal parameters, with a finite probability (as per Bayesian statistics) over and above the randomness of the input datapoints. Truncated time‐domain data, such as SSFP and/or evolution domain interferograms, uniquely benefit from this approach. The linewidth of a signal in a CRAFT simulated spectrum is a function of the measured decay rate constant (in comparison to Fourier transform, which when applied to truncated data, introduces additional broadening)).

CRAFT results are visualized in the conventional spectral format, by first simulating a time‐domain signal (simulated FID) using the parameters dictating each sinusoid, followed by conventional FT. The CRAFT‐FID simulation is not constrained nor dictated by the length (i.e., acquisition time) of the input FID. Typically, the lengths of the simulated FIDs are 3 to 5 times the decay rate constant, thus avoiding any significant truncation artifacts by the subsequent FT, and require no (or nominal, if any) apodization. Moreover, the CRAFT spectra are devoid of any noise. The spectral comparison between CRAFT and SSFP‐FT results in this manuscript are presented such that the analyte peaks are scaled the same to allow direct comparison. As the peaks in CRAFT have improved linewidth, the CRAFT spectra are scaled down by 40 % (i.e. vertical scaling (vs) of 0.6 in each case) to permit this comparison.

In the present study, the combination of SSFP and CRAFT are introduced using ^19^F NMR as an example. Firstly, the approach is demonstrated using a standard mixture, after which it is applied to a range of environmental sample types including tap water, lake water, household paint, biosolids, human serum, and an arctic polar bear liver. The SSFP‐CRAFT combination provides a much more comprehensive evaluation of organic fluorine vs traditional NMR. As it can be applied to any NMR spectrometer or nucleus it holds promise for the analysis of low concentration species in samples across a range of disciplines.

## Results and Discussion

**Figure 1:**
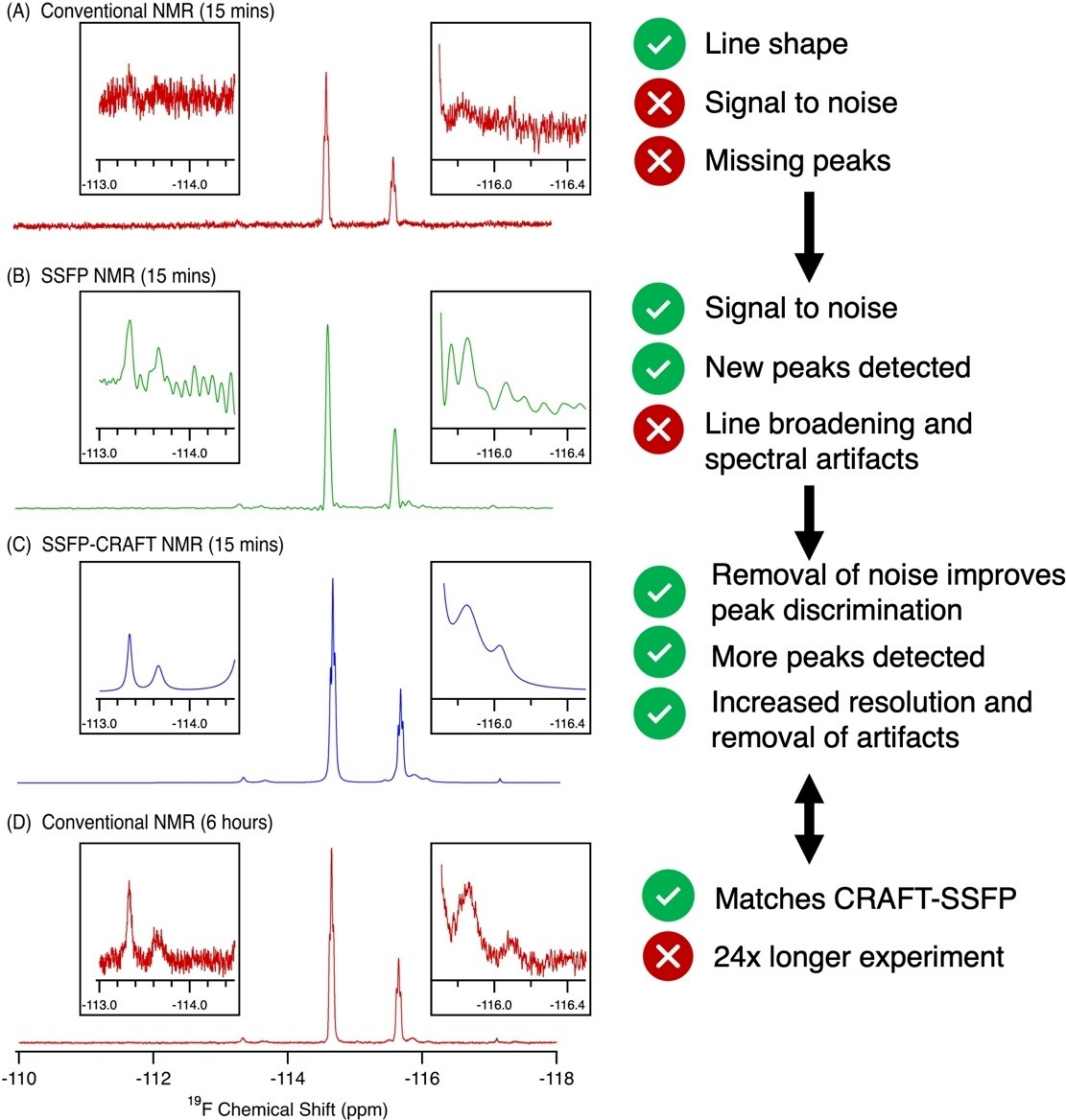
A series of ^19^F NMR spectra demonstrating how the combination of SSFP NMR with CRAFT processing results in a spectrum acquired in only 15 minutes with better signal discrimination, no spectral artifacts, and improved linewidth when compared to a 6 hour conventional NMR experiment. For further analysis of this sample please see supporting Figures S3–S5.

**Figure 2:**
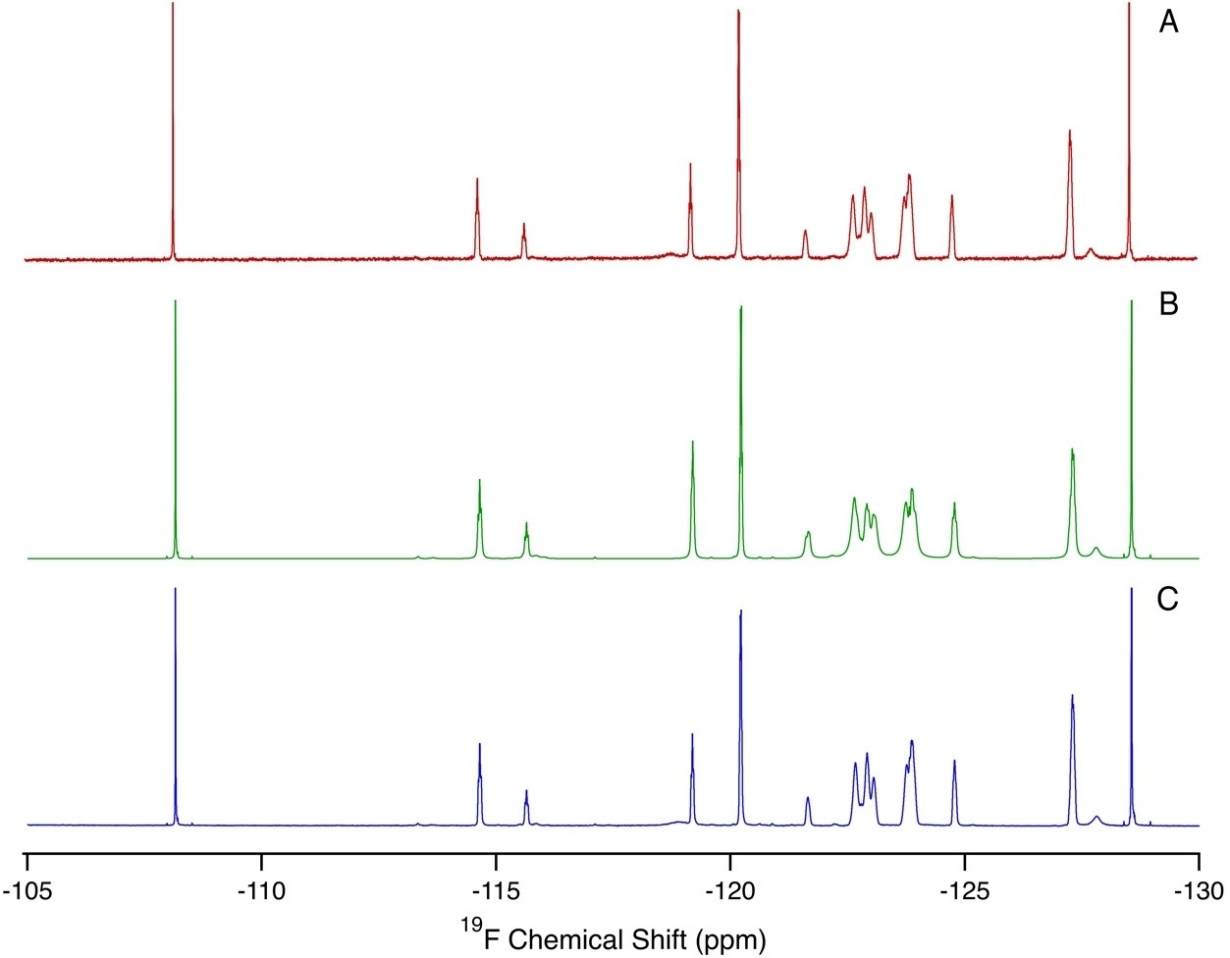
NMR spectra of a mixture of fluorinated compounds highlighting the similarity in relative intensity and line shape between a 14 minute 1D ^19^F NMR experiment (A), a 14‐minute SSFP and CRAFT ^19^F NMR experiment (B), and a 5 hour 39 minute 1D ^19^F NMR experiment (C).

The Supporting Information introduces SFFP, CRAFT and the combined SSFP‐CRAFT approach, including application to a standard sample, discussion of experimental, processing parameters, quantification, and detection limits. For those not interested in the technical details, the overall approach here is summarized in Figures 1 and 2.

Moving from top to bottom; 1A) Standard NMR provides an overview of a sample with excellent line shape, but due to the relatively low detectability of NMR signals, components at low concentration can be missed. 1B) SSFP places the signals in the steady state allowing 10–100’s of scans per second, greatly improving signal‐to‐noise (SNR). Unfortunately, the truncation of the FID introduces artifacts that make discriminating the sample signals challenging. 1C) When CRAFT is used to process the SSFP data, sinusoidal decays are fitted to each signal each with their own linewidth, phase, area and chemical shift, and Fourier transform is avoided. The result is that the spectral features of standard NMR (1A) are recovered while still retaining the increased SNR from SSFP (1B). For comparison, Figure 1D shows the standard NMR of the mixture collected for 6 hours. The results of 1C (collected in only 15 mins) are near identical showing that the combination of SFFP‐CRAFT allows high quality NMR data to be collected very rapidly. In the rest of the manuscript, the combination of SFFP‐CRAFT is demonstrated on a range of high profile biological and environmental samples of considerable scientific importance. While this manuscript focuses on ^**19**^F NMR, the SFFP‐CRAFT combination used here can in principle be applied to *any nucleus in NMR* and as such should find widespread use across many disciplines. Figure 2 highlights the reliability of this approach showing NMR spectra for a mixture of fluorinated compounds including perfluorinated carboxylic acids, perfluoroalkane sulfonates, fluorotelomer alcohols, and perfluorinated ethers, collected using a 14 minute conventional 1D experiment (2A), a 14 minute SSFP‐CRAFT experiment (2B), and a 5 hour 39 minute conventional 1D NMR experiment (2C). This Figure shows that the line shape and relative ratio of signals are similar between the three experiments. This holds true for both high intensity resonances as well as those near the noise level (see Supporting Information Figure S5).

### AFFF‐Impacted Surface Waters

**Figure 3:**
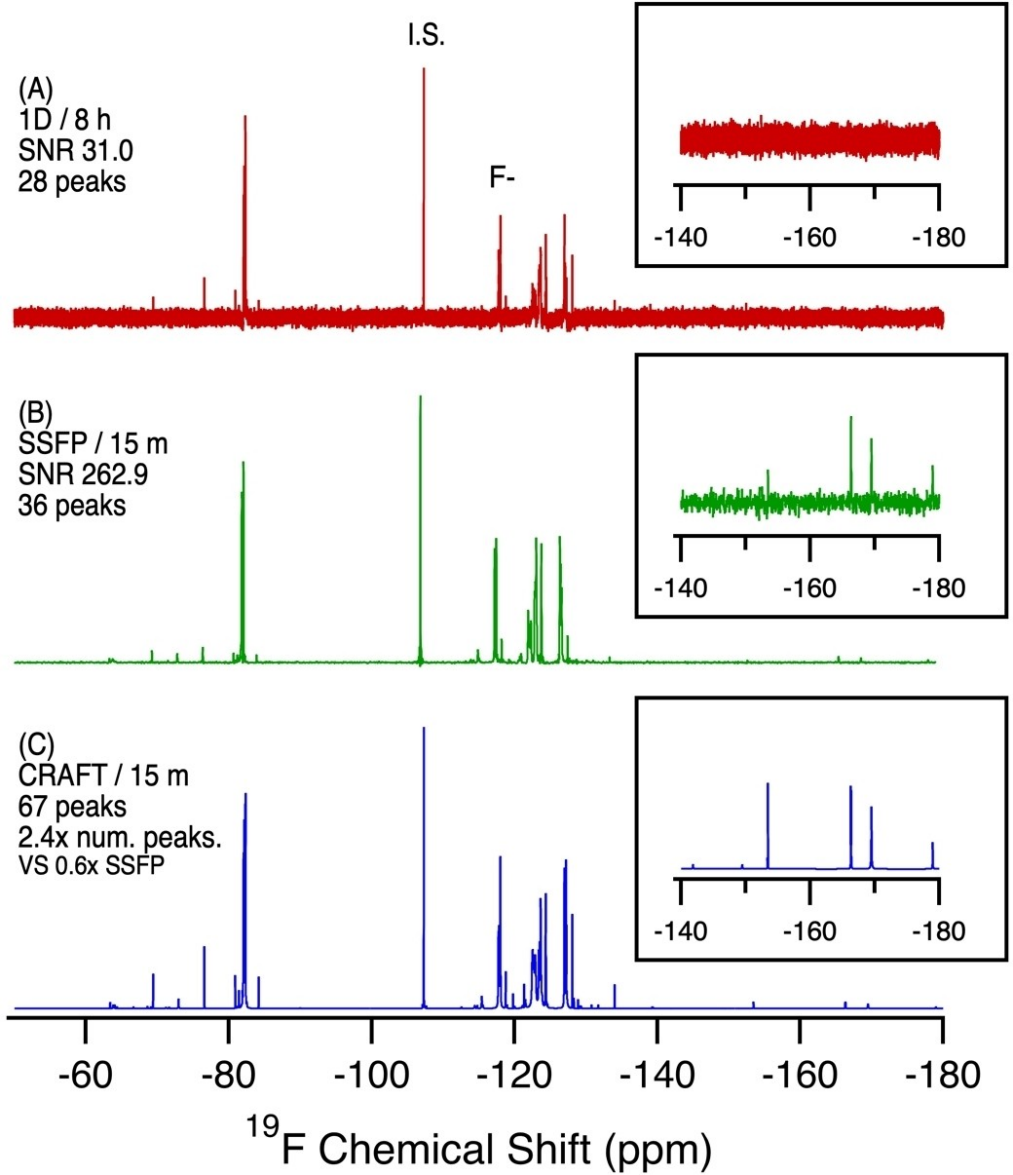
^19^F NMR of Lake Niapenco extract in MeOD‐d_4_ comparing a standard 1D NMR experiment with SSFP and SSFP‐CRAFT. A) Standard 1D NMR, 8 h, 4096 scans. B) SSFP, 15 min, 57,344 scans. SNR calculated from the largest analyte signal (excluding the internal reference standard and F^−^ peak). C) CRAFT processing of B. Note SNR is not provided for the CRAFT data as CRAFT processing eliminates the noise. The number of detectable peaks is a better practical metric for comparison.

Lake Niapenco is known to be contaminated by organofluorine species, thought to be related to upstream aqueous film‐forming foam (AFFF) use.[^22^, ^23^] Figure 3 shows the ^19^F NMR of the pre‐concentrated sample which reveals a wide range of fluorine‐containing compounds present.

Figure 3 shows a comparison between 1D, SSFP, and SSFP‐CRAFT ^19^F NMR of an environmental sample. This Figure clearly demonstrates the exceptional capability of the combined SSFP‐CRAFT method. In just 15 minutes, ^19^F SSFP reveals an additional 8 resonances over the 8‐hour 1D ^19^F experiment. However, signals are partially hidden in the SSFP data due to truncation artifacts that manifest as “wiggles” artificially raising the noise floor, along with spectral broadening, also caused by the FID's truncation. These problems are circumnavigated by CRAFT processing of the SSFP data, which reveals an additional 31 unique resonances over the SSFP results, in the same 15‐minute experiment time. This is a direct result of the improved spectral resolution and enhanced signal to noise ratio available only when SSFP and CRAFT are combined. In‐line with earlier findings from Gauthier and Mabury and de Solla et al.,[^22^, ^23^] chemical shifts were found which correspond to perfluoroalkyl sulfonates (−118 ppm), likely related to perfluorooctane sulfonate (PFOS) which is found in the contaminating AFFF mixture. Additional fluorinated compounds, identifiable only using SSFP with CRAFT, include aryl‐fluorines (−150–−200 ppm) and aromatic trifluoromethyl‐containing compounds (−55–−75 ppm), representative of agricultural and pharmaceutical compounds. Also identifiable are fluorinated branched ether compounds consistent with next generation PFAS such as hexafluoropropylene oxide dimer acid (HFPO‐DA, GenX) and decafluoro‐3H‐4,8‐dioxa‐nonane‐1‐sultonate (ADONA) (δ −83–−90, −130–−145 ppm). With the sharper line shapes produced by the CRAFT data analysis, a clear distinction of 15 unique functional groups with perfluorinated chains is possible (−110–−121 ppm). Such clear distinction of resonances in this critical ^19^F fingerprint region has never been possible before, due to the inclusion of relaxation agents in previous studies.^[22]^

### Drinking Water

**Figure 4:**
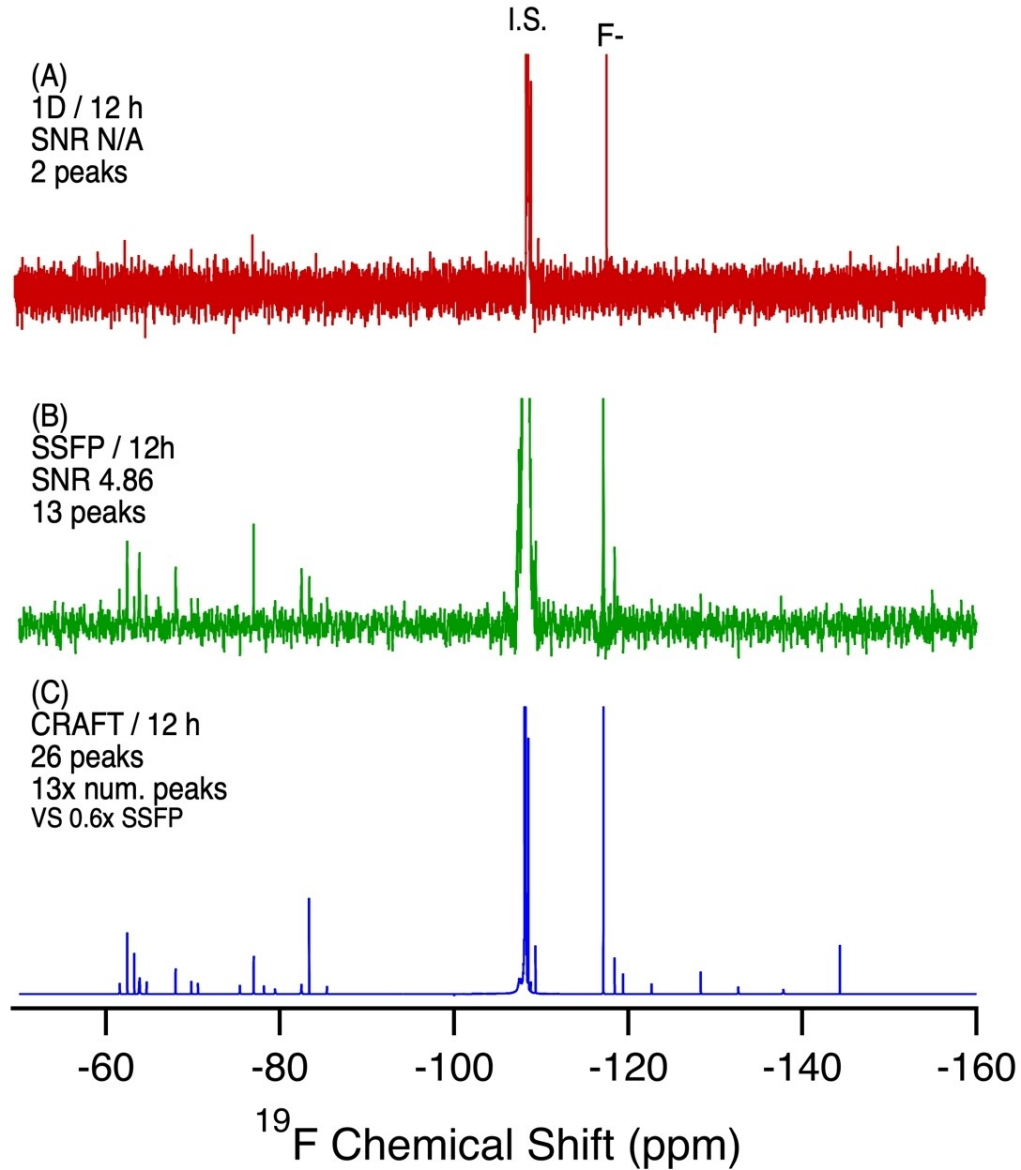
^19^F NMR of Toronto municipal tap water extract in MeOD‐d_4_ (4L by WAX and HLB SPE) comparing a standard 1D NMR experiment with SSFP and SSFP‐CRAFT. A) 1D NMR, 12 h, 6656 scans. B) SSFP, 12 h, 2,621,440 scans. C) CRAFT processing of B. SNR calculated from the largest analyte signal (excluding the internal standard and F^−^ peak).

In drinking water samples, the presence of PFAS is of great concern as their occurrence can be directly correlated with a rise in PFAS human serum levels.[^24^, ^25^, ^26^] However, the low levels present are an analytical challenge for any method. In tap water from Toronto, Canada, which serves >1 million people, conventional ^19^F NMR (even after 12 hrs) only shows signals from the internal standard and fluoride (see Figure 4A). However, with SSFP (4B) 11 additional resonances are revealed. These correspond to the presence of perfluorinated carboxylic acids (PFCAs, −82, −119 ppm), trifluoroacetic acid (TFA, −77 ppm), and interestingly, several aromatic trifluoromethyl compounds (−61.5, −62.4, −63.8, −68.1 ppm). CRAFT analysis of the SSFP data further reveals a multitude of additional aromatic‐CF_3_ resonances (−60–−70 ppm), additional resonances related to the presence of PFAS including short‐chain ether acids (−70–−80 ppm) and resonances between −130 and −150 ppm, which typically corresponds with branching polyfluorinated structures or branching ether acids, such as hexafluoropropylene oxide dimer acid (HFPO‐DA) (−130–−150 ppm), as well as aryl‐fluorine compounds at resonances between −150 and −200 ppm.^[22]^

The presence of the aromatic compounds, only revealed using SSFP and CRAFT, is of significant interest. Typical analysis of fluorinated compounds is performed by mass spectrometry, with a focus on the analysis of anionic PFAS. These routine approaches would normally not detect these aromatic compounds. However, the ^19^F NMR data suggests the most significant contribution to the total fluorine mass balance outside of inorganic fluoride, are aromatic‐CF_3_ compounds which are generally associated with agricultural or pharmaceutical compounds. This raises important questions as to the extent to which the human population experiences unintentional exposure to pharmaceuticals through drinking water sources.

### House Paint

**Figure 5:**
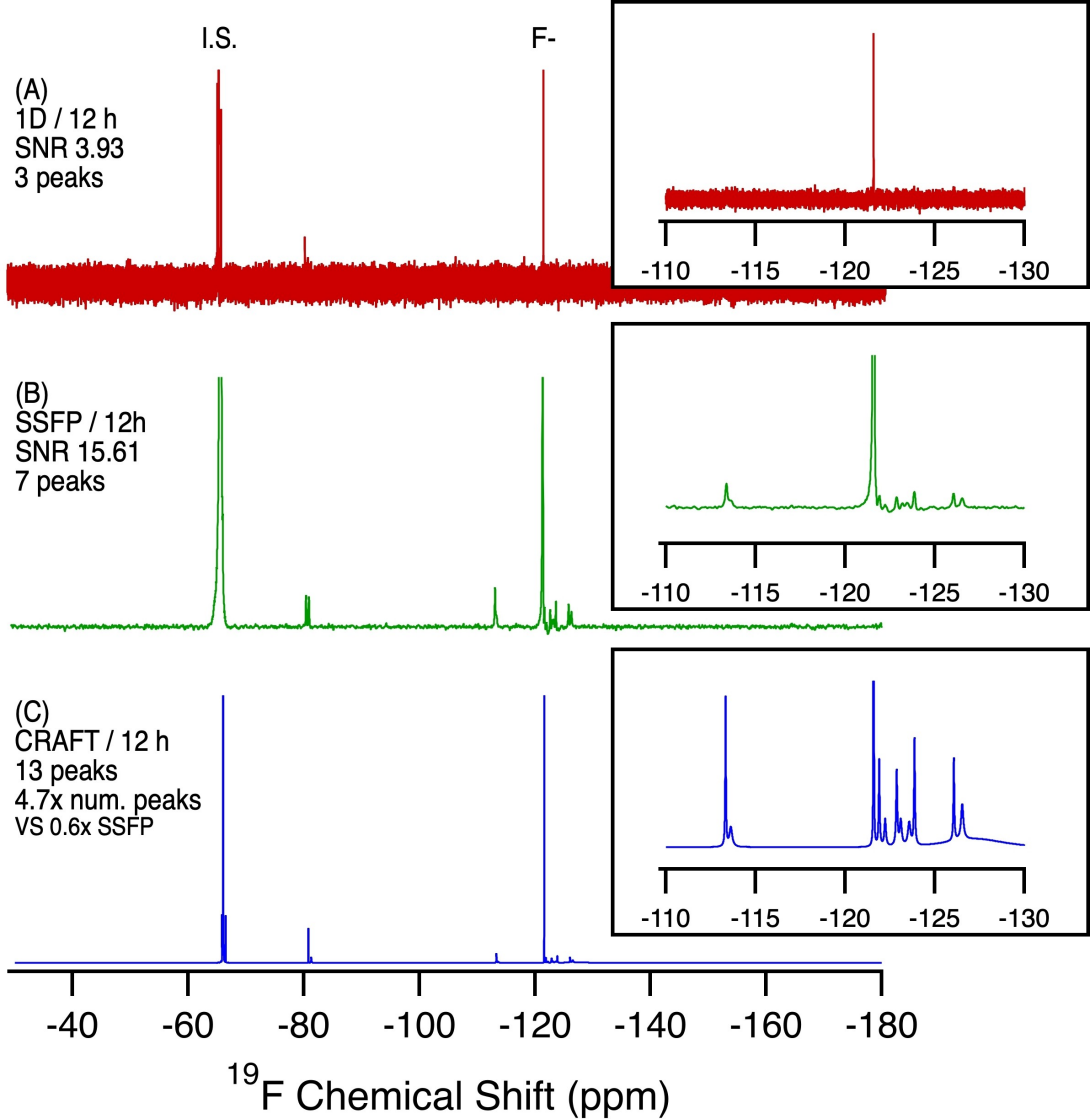
^19^F NMR of an exterior house paint sample. 100 mg of house paint was dissolved in D_2_O and analyzed by standard 1D NMR, SSFP, SSFP‐CRAFT. A) Standard 1D NMR, 12 h, 6656 scans. B) SSFP, 12 h, 2,621,440 scans. C) CRAFT processing of B. SNR calculated from the largest analyte signal (excluding the internal standard and F^−^ peak).

A study by Cahuas et al. used a high field instrument (800 MHz) and cryogenically cooled probe to directly measure total fluorine in house paints by ^19^F NMR. Several paint samples were found to contain the 6 : 2 fluorotelomer alcohol (6 : 2 FTOH).^[27]^ The question becomes, can a room temperature NMR probe, at 500 MHz (~1/5^th^ of the cost) achieve similar results if SSFP and CRAFT are combined. A conventional 1D NMR experiment (Figure 5A) reveals only inorganic fluoride (−121.4 ppm), and a small terminal alkyl‐CF_3_ resonance (−81 ppm) which corresponds to perfluorinated alkyl chains of two carbons or greater.[^12^, ^22^, ^28^] The use of SSFP (Figure 5B) reveals an additional 4 resonances, including the unique resonance for the CF_2_ nearest a fluorotelomer alcohol group (−113.4 ppm), an additional terminal alkyl‐CF_3_ resonance at −81.4 ppm, as well as resonances corresponding to perfluorinated alkyl‐CF_2_ chains (−121–−126 ppm). The CRAFT processed data with higher spectral resolution reveals additional detail, including multiple fluorotelomer species, a similar finding to the Cahuas study. This example demonstrates the improvement in detectability of compounds on more commonly available instrumentation as well as the potential for NMR spectroscopy to perform direct analysis of low concentration compounds in complex samples where other analytical techniques would be challenging due to matrix effects.

### Polar Bear Liver

**Figure 6:**
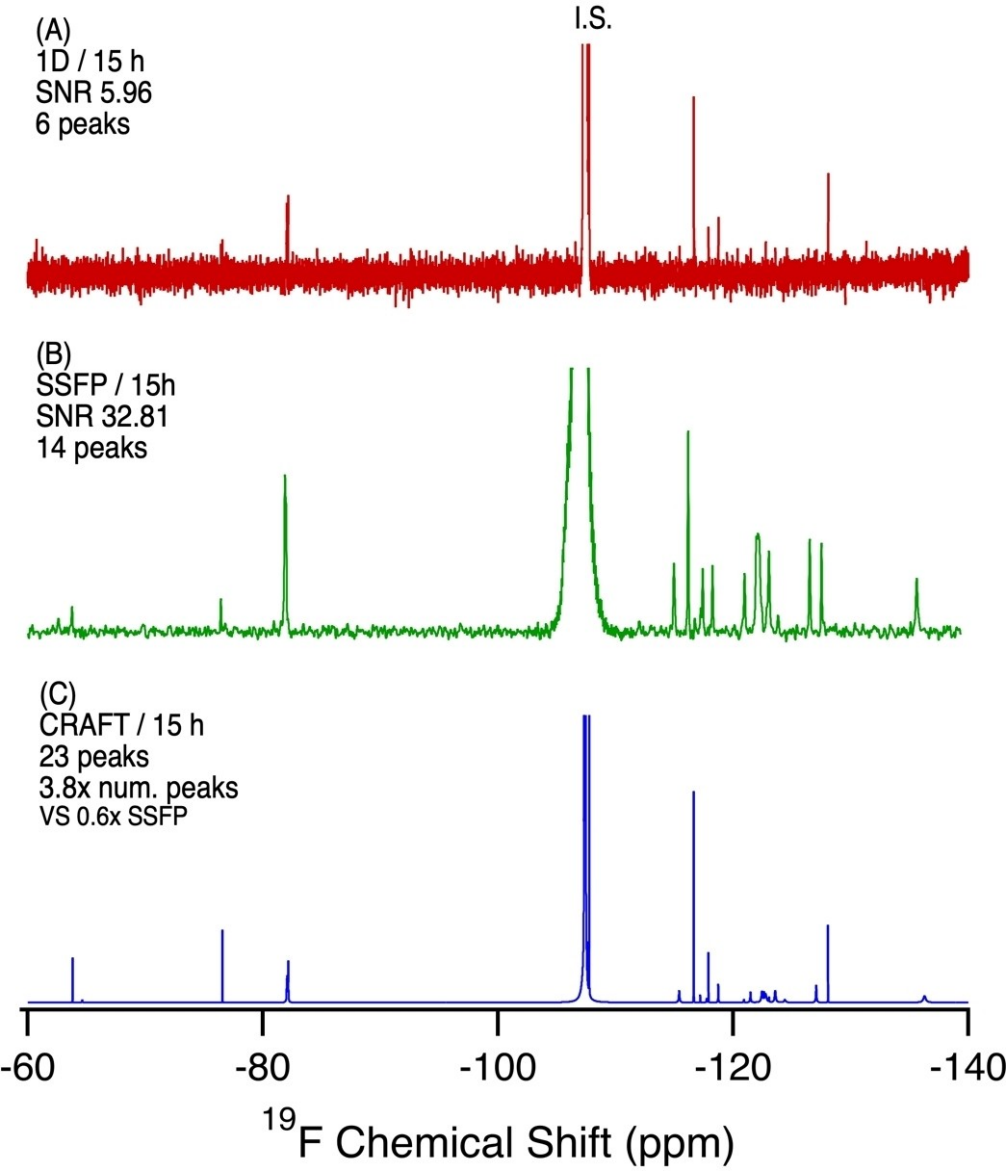
^19^F NMR of an archived polar bear liver sample from the Southern Hudson Bay population collected in 2005. Sample preparation was a simple homogenization of the liver (100 mg) in deuterated methanol, centrifugation, and transfer of the supernatant to the NMR tube. A) 1D NMR, 15 h, 6144 scans. B) SSFP, 15 h, 2,097,152 scans. C) CRAFT processing of B. SNR calculated from the largest analyte signal (excluding the internal standard and F^−^ peak).

The detection of PFAS in polar bears has considerable impacts for understanding global transport, biomagnification, and transfer via food chains, given that polar bears are not exposed to any point sources of PFAS.[^3^, ^27^] Figure 6 shows the ^19^F NMR results from direct extraction of 100 mg of the polar bear liver. Standard 1D ^19^F NMR shows signals consistent with perfluorinated carboxylic acids, albeit with low SNR. The CRAFT analysis of the SSFP data offers significant additional information. At least six different functional group chemistries are present in the CRAFT spectra, evidenced by the unique CF_2_ resonances found between −115 and −120 ppm. These resonances correspond with the CF_2_ nearest the polar functional group on fluorinated alkyl chains and can be used for tentative identification of PFAS in this sample, including the PFCAs (−118.7 ppm), PFSAs (−117.9 ppm), sulfonamides (−117.7, −117.2 ppm), and fluorotelomer species (−116.6, −115.5 ppm). Of interest is the presence of both TFA (−77 ppm) and aromatic‐CF_3_ compounds (−63.8, −64.6 ppm). While TFA has been shown to undergo long range transport, it is not expected to bioaccumulate in polar bears and is therefore surprising to find in polar bear liver at elevated levels. Aromatic‐CF_3_ compounds, as previously discussed, are likely to be related to agricultural or pharmaceutical chemistries. Given that polar bears are not exposed to any point sources of these compounds, it raises an important research question as to how these fluorinated compounds arrived in the Arctic.

### Biosolids

**Figure 7:**
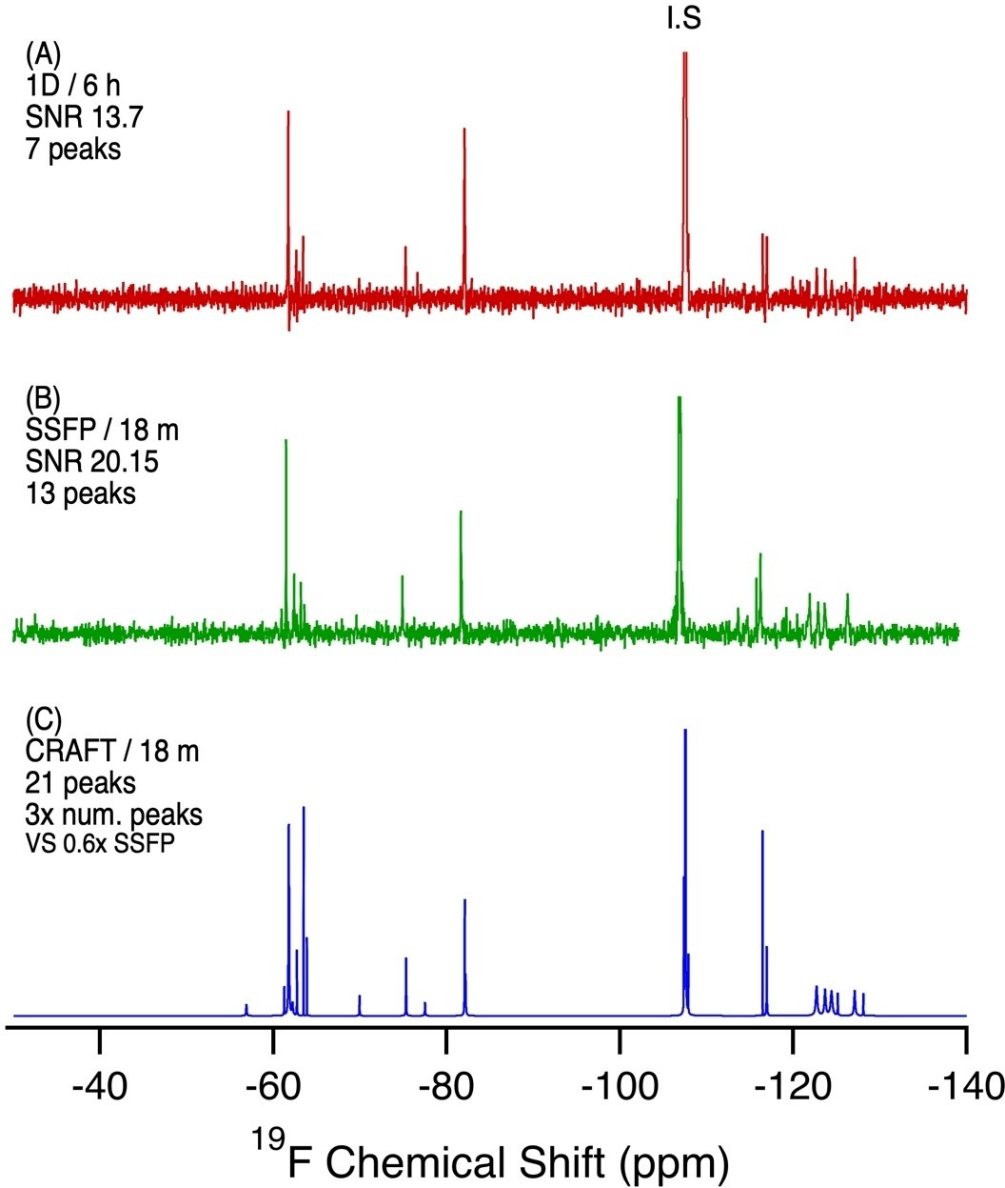
^19^F NMR of a biosolid (35 g) extract in MeOD‐d_4_. Sample preparation was a simple methanol extraction. A) 1D NMR, 6 h, 3200 scans. B) SSFP, 18 min, 65,536 scans. C) CRAFT processing of B. SNR calculated from the largest analyte signal (excluding the internal standard and F^−^ peak).

Biosolids prepared from wastewater treatment plants are an excellent metric for exposure of the general population to PFAS and other chemicals. The biosolids analyzed in the present study were prepared from sedimentation tanks at a Toronto, Canada wastewater treatment plant, which serves over 1.5 million people in a dense urban setting. Biosolids are interesting also in that they are mostly used as agricultural fertilizer. This raises concerns regarding recycling of fluorinated compounds back into the human population through food production.^[30]^ The 1D ^19^F NMR experiment in the present study (Figure 7A) contains several fluorine resonances at reasonable SNR. This suggests a reasonably high concentration of fluorine‐containing compounds in these biosolids. Letcher et al.^[31]^ found high concentrations of side‐chain fluoropolymer surfactants in Canadian wastewater treatment plants. These fluorinated compounds could help explain the high concentrations of fluorine‐containing compounds with sulfonamido‐type chemistry present in this sample. Resonances corresponding to the PFCAs and PFSAs are present, as well as TFA. Notably, the most intense resonances correspond to aromatic‐CF_3_ compounds, which is not unexpected given the high use of pharmaceuticals containing aromatic‐CF_3_ groups, and high occurrence of pharmaceuticals in wastewater treatment plants.[^32^, ^33^, ^34^] The SSFP results (Figure 7B) show additional resonances in this aromatic‐CF_3_ region, while processing with CRAFT (Figure 7C) reveals the true extent of contamination from fluorine‐containing pharmaceuticals. Both the SSFP and CRAFT methods show more spectral detail in an 18‐minute experiment than the 1D experiment achieves in 6 hours. There are an additional 5 resonances in the aromatic‐CF_3_ region, one of which is broad and intense, suggesting many overlapping resonances, likely of pharmaceutical origin. This result shows the capability of ^19^F NMR to provide a fingerprint type overview of the types of PFAS in the sample.

### Human Serum

**Figure 8:**
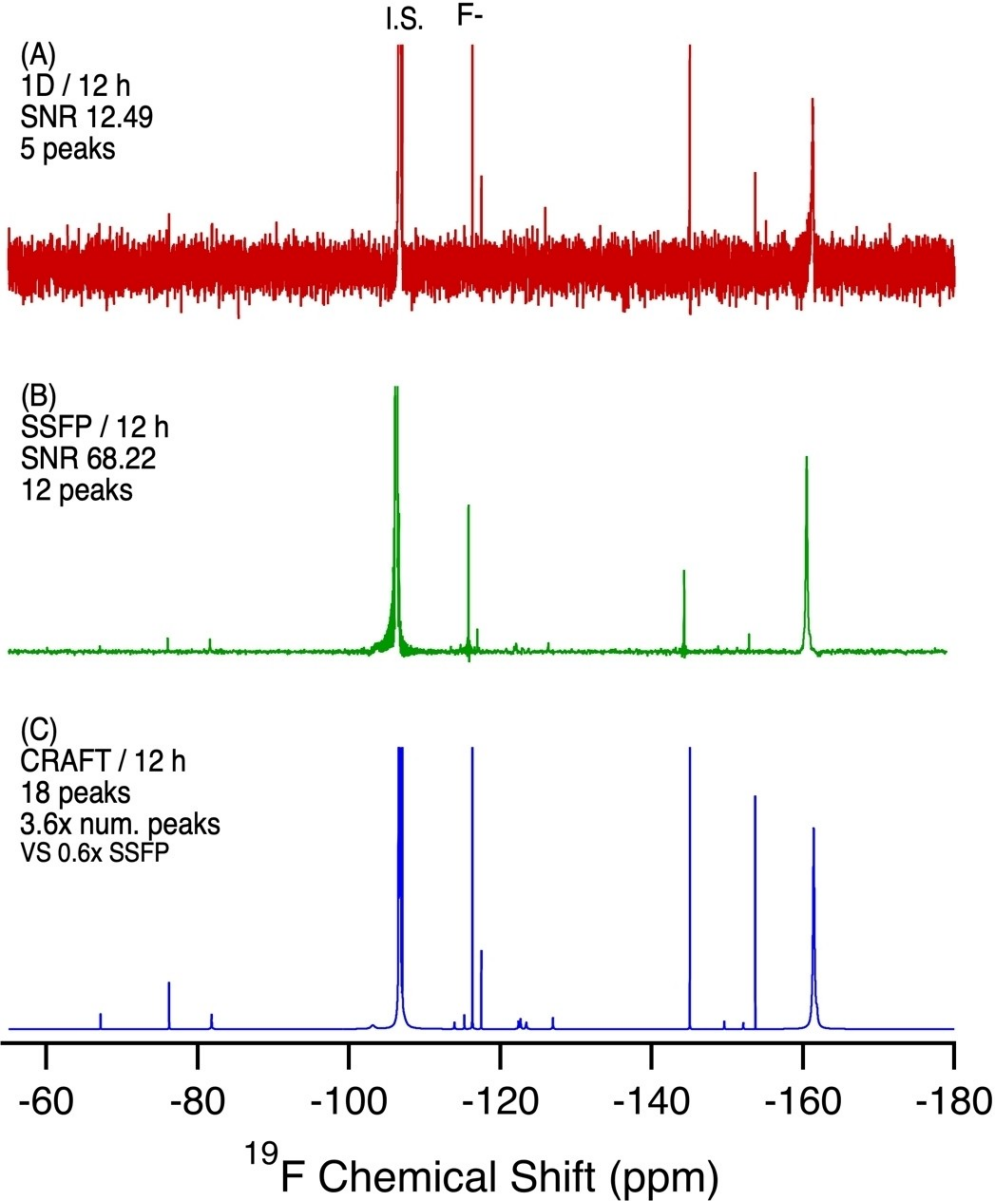
^19^F NMR of human serum (1.0104 g) extracted in MeOD‐d_4_. A) 1D NMR, 12 h, 6451 scans. B) SSFP 12 h, 2,539,520 scans. C) CRAFT processing of B. SNR calculated from the largest analyte signal (excluding the external standard and F^−^ peak).

Human serum is challenging to analyse by techniques which are subject to high matrix effects, such as LC‐MS. Typical methods involve using an ion‐pairing agent to remove charged fluorine‐containing species such as anionic PFAS from the matrix.^[35]^ However, the potential to miss the plethora of neutral PFAS and pharmaceuticals which are expected to be found in the human population is high.[^11^, ^35^] In this example, just 1 g of serum was extracted in methanol and analysed as is. Figure 8 highlights the additional information gained from both the SSFP and CRAFT methods over the standard 1D ^19^F NMR experiment.

Using CRAFT and SSFP, the human serum sample was shown to contain several PFAS‐related resonances including fluorotelomer compounds (−113.9, −115.2 ppm), PFCAs, and PFSAs. Interestingly, TFA was present as a very intense resonance. As TFA is not thought to be bioaccumulative in humans, its presence in this pooled serum sample at apparent elevated levels is of significant interest. This raises an important question as to whether TFA is accumulating in serum, or if the presence of TFA in serum represents a base‐level concentration to which this population has been exposed, or if TFA is being formed through some other mechanism in the blood or serum. Also present are resonances of significant intensity corresponding to aryl‐fluorine chemistry (−149.6, −152.2, −153.7, −161.4 ppm) which can be attributed to pharmaceuticals/agrochemicals. While there is significant PFAS in this sample, notably of fluorotelomer species, the predominant fluorine mass balance appears to be derived from pharmaceutical compounds.

### Detection Limits and Quantification

Supporting Figure S8 investigates a human blood example, determining the detection limits of PFOA spiked directly into human whole blood. At 50 μM in 15 hr, standard NMR can just detect the analyte at ~3 : 1 SNR (i.e. at the limit of detection). Conversely, SSFP‐CRAFT is still able to recover signal from the main CF_2_ chain with 10 fluorine nuclei at 1 μM resulting in a very impressive ~50‐fold increase in detection limit. This example is further discussed in the Supporting Information and highlights the ability of SSFP‐CRAFT to lower detection limits in highly complex samples, allowing for direct analysis in some examples.

The main limitation of the combined SSFP‐CRAFT approach at present is that the technique is not fully quantitative. Larger species such as polymers and macromolecules will relax faster than small, fluorinated compounds, resulting in the acquisition of more signal per scan. To investigate the quantitative potential of SSFP‐CRAFT, several NMR experiments were performed on a known mixture of six fluorinated compounds with varying physiochemical properties and functional group chemistry. This sample was also examined using LC‐MS/MS. The full details for this experiment are provided in the Supporting Information section 1.7. Notably, the inclusion of paramagnetic relaxation agent chromium acetylacetonate (Cr) as well as increasing the length of the FID (SSFP acquisition time) while maintaining the steady state regime led to an increase in the accuracy of the quantitative spike and recovery experiment. With a 200 ms acquisition window and 8 mg/mL Cr, the average percent recovery (*n*=3) was 97 % with the largest error less than 10 % even in these preliminary tests.

This suggests the method can be made fully quantitative but experimental conditions (i.e. ideal concentration of relaxation agent, type of relaxation agent, and acquisition time) should be explored further for optimal results. Notably, when compared to the MS results, NMR and SSFP‐CRAFT was better able to quantify those compounds which are not readily amenable to LC separation and ionization techniques, highlighting the benefit of using NMR as a complimentary tool for MS based analysis.

## Conclusion

In this study, we demonstrate the applicability of SSFP NMR with CRAFT processing for the assessment of PFAS and other fluorinated chemicals using solution‐state NMR spectroscopy. In every example, the combination of SSFP and CRAFT reveals additional resonances of interest, and consistently demonstrates an increase in SNR and improved limit of detection. The increase in SNR has provided a more complete picture of fluorine contamination in environmental and biological samples than has been possible before. ^19^F SSFP‐CRAFT NMR represents a versatile and powerful tool to detect persistent fluorinated contaminants at trace levels in solution, many of which cannot be observed without the SSFP‐CRAFT approach. The experiment and analysis are straightforward, takes no additional experimental time when compared with conventional 1D NMR, and can be performed on any NMR spectrometer. In fact, as demonstrated often, experiments can be significantly shortened while retaining high quality NMR data. Despite the need for further optimization of quantitation, even in its current state SSFP‐CRAFT improves the limit of detection of all components, leads to the discovery of many new resonances, and improves the detection limits of PFOA in human blood by ~50 times. In a standard mixture, the approach detected compounds missed by LC‐MS/MS. SSFP‐CRAFT holds great potential as a discovery tool to complement more commonly used MS based methods. In summary, as SSFP‐CRAFT can be applied to any nucleus it holds promise across many areas of science, from analytical chemistry, through environmental monitoring, to human health.

## Supporting Information

The Supporting Information is available free of charge online. It includes additional details on implementing the SSFP and CRAFT experiments, discussion of CRAFT model parameters, sample preparation details, limits of detection, and limitations of the approach. The authors have cited additional references within the Supporting Information.[^13^, ^14^, ^15^, ^22^, ^36^, ^37^]

## Conflict of Interests

The authors declare no conflict of interest.

1

## Supporting information

As a service to our authors and readers, this journal provides supporting information supplied by the authors. Such materials are peer reviewed and may be re‐organized for online delivery, but are not copy‐edited or typeset. Technical support issues arising from supporting information (other than missing files) should be addressed to the authors.

Supporting Information

## Data Availability

The data that support the findings of this study are available from the corresponding author upon reasonable request.
