# Supplementary material for: Steady State Free Precession NMR without Fourier Transform: Redefining the Capabilities of 19F NMR as a Discovery Tool
Source: Angew Chem Int Ed Engl. 2025 Feb 10;64(15):e202422971. doi: 10.1002/anie.202422971 (PMC11976210; doi:10.1002/anie.202422971)
Supplement: Supplementary file 1 — Supporting Information [file ANIE-64-e202422971-s001.pdf]

Angewandte Chemie
Eine Zeitschrift der Gesellschaft Deutscher Chemiker

Supporting Information

Steady State Free Precession NMR without Fourier Transform: Redefining the Capabilities of ^{19}F NMR as a Discovery Tool

J. R. Gauthier, F. Kock, K. Downey, T. Moraes, L. S. Almeida, D. C. G. Muir, R. J. Letcher, L. Colnago, K. Krishnamurthy, S. Mabury, A. J. Simpson**

Steady-state free precession NMR without Fourier transform: Redefining the capabilities of ^{19}F NMR as a discovery tool

Jeremy R. Gauthier[a] †, Flavio Kock[b,c] †, Katelyn Downey[b], Tiago Moraes[d], Luísa Souza Almeida[e], Derek C. G. Muir[f], Robert J Letcher[g], Luiz Colnago[h], Krish Krishnamurthy[i], Scott Mabury[a]*, Andre J Simpson[a,b]*

† Both authors equally collaborated for this work

[a] Department of Chemistry, University of Toronto, 80 St George Street, Toronto, ON, M5S 3H6, Canada.

[b] Physical and Environmental Sciences, University of Toronto Scarborough, 1265 Military Trail, Toronto, ON, M1C 1A4, Canada.

[c] Departamento de Ciencias – Química, Centro de Espectroscopia de Resonancia Magnética Nuclear (CERMN), Pontificia Universidad Católica del Perú, Av. Universitaria 1801, Lima 32, Lima, Peru.

[d] Department of Biosystems Engineering, São Paulo University, 11 Av. Páduas Dias, Piracicaba, SP, 13418-900, Brazil.

[e] São Carlos Chemistry Institute, São Paulo University, 400 Av Trabalhador São-Carlense, São Carlos, SP, 13566-590, Brazil.

[f] Canada Centre for Inland Waters, Environment and Climate Change Canada, 867 Lakeshore Rd, Burlington, ON, L7S 1A1, Canada.

[g] National Wildlife Research Centre, Environment and Climate Change Canada, 1125 Colonel By Drive (Raven Road), Carleton University, Ottawa, ON, K1A 0H3, Canada

[h] Embrapa Instrumentation, 1452 Rua XV de Novembro, São Carlos, SP, 13560-970, Brazil.

[i] Krish Krishnamurthy, Chempacker LLC, 3054 Beckley drive, CA 95135, USA

*Corresponding authors: scott.mabury@utoronto.ca, andre.simpson@utoronto.ca

1 Discussion of SSFP, CRAFT and the combined SSFP-CRAFT approach

1.1 Description of the SSFP pulse sequence **2**

1.2 Optimizing SSFP experiment parameters **3**

1.3 Combining CRAFT and SSFP **6**

1.4 Discussion of CRAFT model parameters **11**

1.5 Limits of detection in complex samples **15**

1.6 Method Limitations **16**

1.7 Investigation of the potential for SSFP to perform quantitative measurements **17**

2 Experimental

2.1 Sample preparation **21**

2.2 NMR Experimental parameters **24**

2.3 Data processing **25**

3 Pulse Code and Processing Scripts

3.1 ^{19}F SSFP Pulse program code **27**

3.2 Macros for processing SSFP data in TopSpin by Fourier transform **29**

Section 1 – Discussion of SSFP, CRAFT and the combined SSFP-CRAFT approach

1.1 Description of the SSFP Pulse Sequence

Signal-to-noise ratio (SNR), spectral resolution, and the acquisition time are factors which can influence the applicability of NMR spectroscopy for the measurement of trace components in complex environmental and biological systems. The steady-state free precession (SSFP) pulse sequence can boost the sensitivity of NMR, allowing for measurement of compounds at lower concentrations not achievable using standard 1D NMR experiments.

Nuclei can be placed into a steady state by applying a train (n) of pulses with optimized flip angle (Θ_1). The delay between pulse T_p (AQ + recycle delay) is much less than the T2* of the sample. This setup is illustrated in Figure S1. T_2* corresponds to the time it takes for the FID to decay to zero and is dependent on the transverse relaxation time (T_2) and the magnetic field inhomogeneity. The resultant intensity for the NMR signals obtained using SSFP does not have dependence on the longitudinal relaxation time (T_1), resulting in rapid accumulation of scans due to the faster relaxation time dependence versus standard 1D NMR pulse sequences. The result is the accumulation of thousands of scans in very short experiment times, and higher SNR in comparison with conventional NMR experiments. The additional pulse Θ_x and corresponding delay T_{purge} are an optional purge block used to cancel the standard NMR signal such that only the steady state signal is recorded.

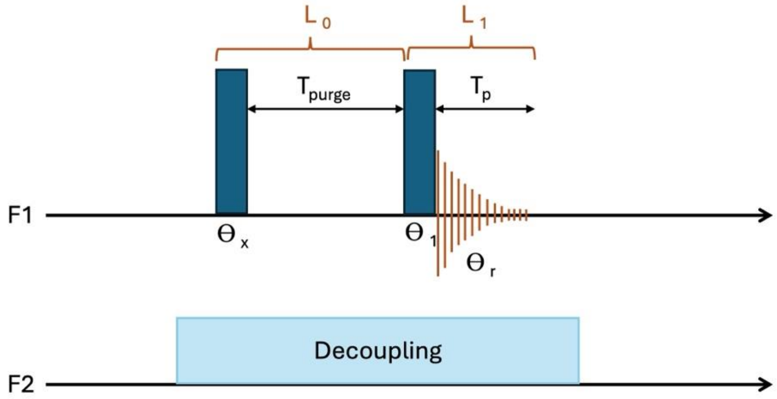

Figure S1. SSFP pulse sequence; pulses are applied with an optimized flip angle and T_p (sum of the acquisition time and recycle delay) much less than the transverse relaxation time. L_1 is the number of scans and L_0 is an optional PURGE block to cancel non-steady-state magnetization. In the SSFPd4[1] sequence used here L_0 and L_1 blocks are incremented 4 times with Φ_1 and Φ_r are phase cycled as previously described. The full pulse program is provided in Section 3.1 of this Supporting Information.

1.2 Optimizing SSFP experiment parameters

The SSFP pulse sequence does not normally use a 90° RF pulse. The exact pulse length is highly dependent on the sample and should be calibrated for each sample, with goal to attain maximum signal per unit time. The efficiency of the SSFP pulse sequence varies greatly depending on the sample composition. As discussed in the main text of the manuscript, smaller molecules in cleaner matrices have longer relaxation rates. If the sample is suspected to contain small molecules in clean matrices, such as the case in the analysis of reference standards or samples extracted for mass spectrometry, using a short pulse width will help compensate for the longer relaxation time and prevent "saturation" of the steady-state signal. The result, however, is only a small portion of the equilibrium magnetization of the sample being detected. In contrast, larger molecules or more complex sample matrices can take advantage of a longer pulse width, as they relax faster, and it is possible to retain more magnetization in the steady state. As such, signals with fast T_2 are emphasized by steady state NMR. To demonstrate this let's consider two extremes. At one extreme, a very small molecule with very fast tumbling (for example the fluoride ion in the

drinking water (Figure 4) will have a very long relaxation time. With an acquisition time of only 29 ms it is feasible only 10% of its equilibrium magnetization is detected per pulse. Because so many pulses are possible per unit using SSFP even in this worst-case scenario SSFP still improves SNR by more than two times. However, if we now consider a polymer, in comparison it will have much faster relaxation. It is feasible that in the same 29 ms acquisition time the equilibrium state signal is fully recovered. In this example the polymer would be seen 10x better in comparison to the fluoride ion. Ultimately, SSFP will always bias the signals that have fast relaxation. Arguably, this is highly complementary to standard NMR. While fully quantitative NMR sees all spins the same in terms of signal area, faster relaxing components such as polymers have broader linewidths, which in turn means they can be harder to distinguish in mixtures, especially when close to the noise level and overlapping with other peaks. As such the ability of SSFP to bias these otherwise "harder to detect" components make it an important and complementary tool.

Optimization of the pulse width, or flip angle (ϴ), for the SSFP experiment is best performed empirically. A series of SSFP experiments should be acquired using a reference standard or representative sample which allows for observation of the signal in a reasonable number of scans. If a standard is used it should be either spiked into the sample of interest or spiked into an identical matrix. A series of short SSFP experiments can be used with a flip angle (ϴ) beginning at 5° and increased in stepped increments of 5° until 90°. This is demonstrated in Figure S2, which shows the relative signal intensity of the alkyl-CF_3 resonance within the Lake Niapenco and human serum samples at increasing flip angles. In this example, a maximum signal intensity is reached between flip angles (ϴ) of 45° and 50° for Lake Niapenco, while the flip angle maximum is 65° for the more complex human serum sample. An identical process was used to calibrate the SSFP flip angles for all other samples, results from which are included in Table S1.

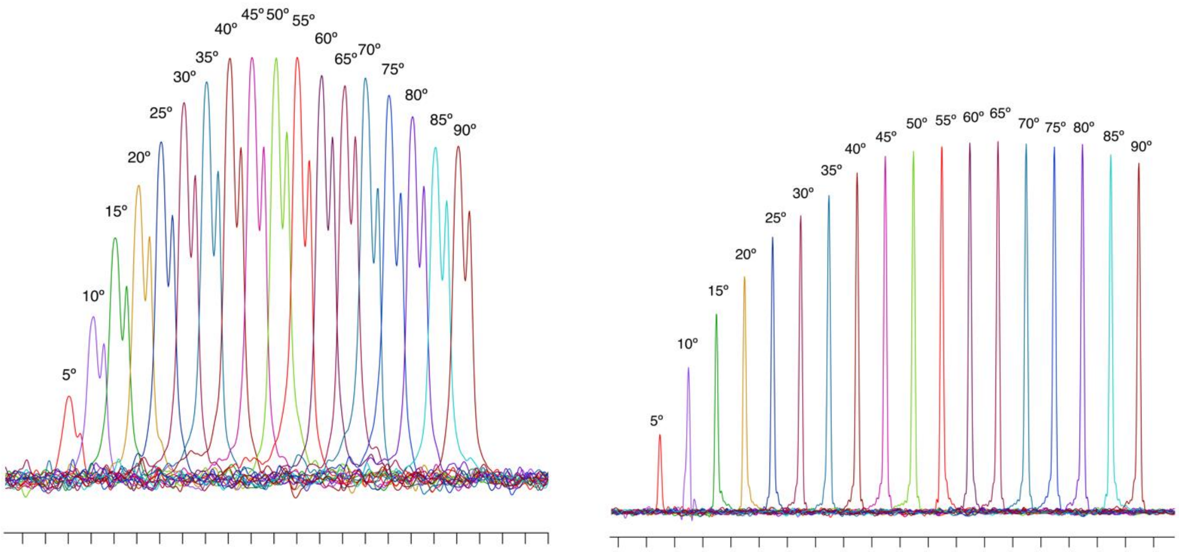

Figure S2: ^{19}F SSFP NMR optimization of the alkyl-CF_3 resonance (δ -80.7 ppm) in the Lake Niapenco water sample (left) and in the human serum sample (right). Flip angle (ϴ) in degrees is indicated above each resonance which corresponds to increasing pulse length. All resonances are -80.7 ppm, the peaks are offset by 0.4 ppm to clearly show the change in relative signal intensity. The y-axis shows the relative intensity between each resonance in each sample.

Table S1: Optimized SSFP acquisition parameters for each sample discussed in this study.

**Sample**	**Pulse Width (μs)**	**Flip Angle (ϴ)**
Lake Niapenco	3.93	45°
Drinking Water	5.33	75°
House Paint	2.14	35°
Polar Bear Liver	5.42	65°
Biosolids	5.75	65°
Human Serum	4.95	65°

1.3 Combining CRAFT and SSFP

By maintaining nuclei in the “steady-state” the loss of signal from this state is continuously sampled potentially allowing for thousands of scans in the same time as a single standard 1D NMR scan, in turn boosting sensitivity. This is best demonstrated through example. Figure S3 compares conventional ^{19}F NMR, SSFP ^{19}F NMR, and SSFP-CRAFT ^{19}F NMR on a standard sample.

In Figure S3A, the 1D NMR spectrum has relatively low SNR which is more apparent for signals in the inset (110-118 ppm). SSFP acquired in the same amount of time improves the detection limits. Direct comparison to the SSFP spectrum in Figure S3B highlights that while many resonances are identifiable in the 1D NMR, additional information is gained by the increased SNR available from the SSFP experiment. In non-targeted screening methods, such as the use of NMR in the present study, it is important to ensure all potential information is gained from a sample. The increased sensitivity offered by SSFP helps in this regard, revealing additional resonances which are otherwise beneath the noise in standard 1D NMR experiments. However, the increased sensitivity comes with additional limitations, namely the introduction of line broadening and artifacts which are a result of the highly truncated free induction decay. As shown in the inset in Figure S3B, while new resonances are visible, also visible is the appearance of truncation artifacts surrounding the more intense resonances, and “wiggles” adding to the noise floor. It is not easy to differentiate between these artifacts and real peaks.

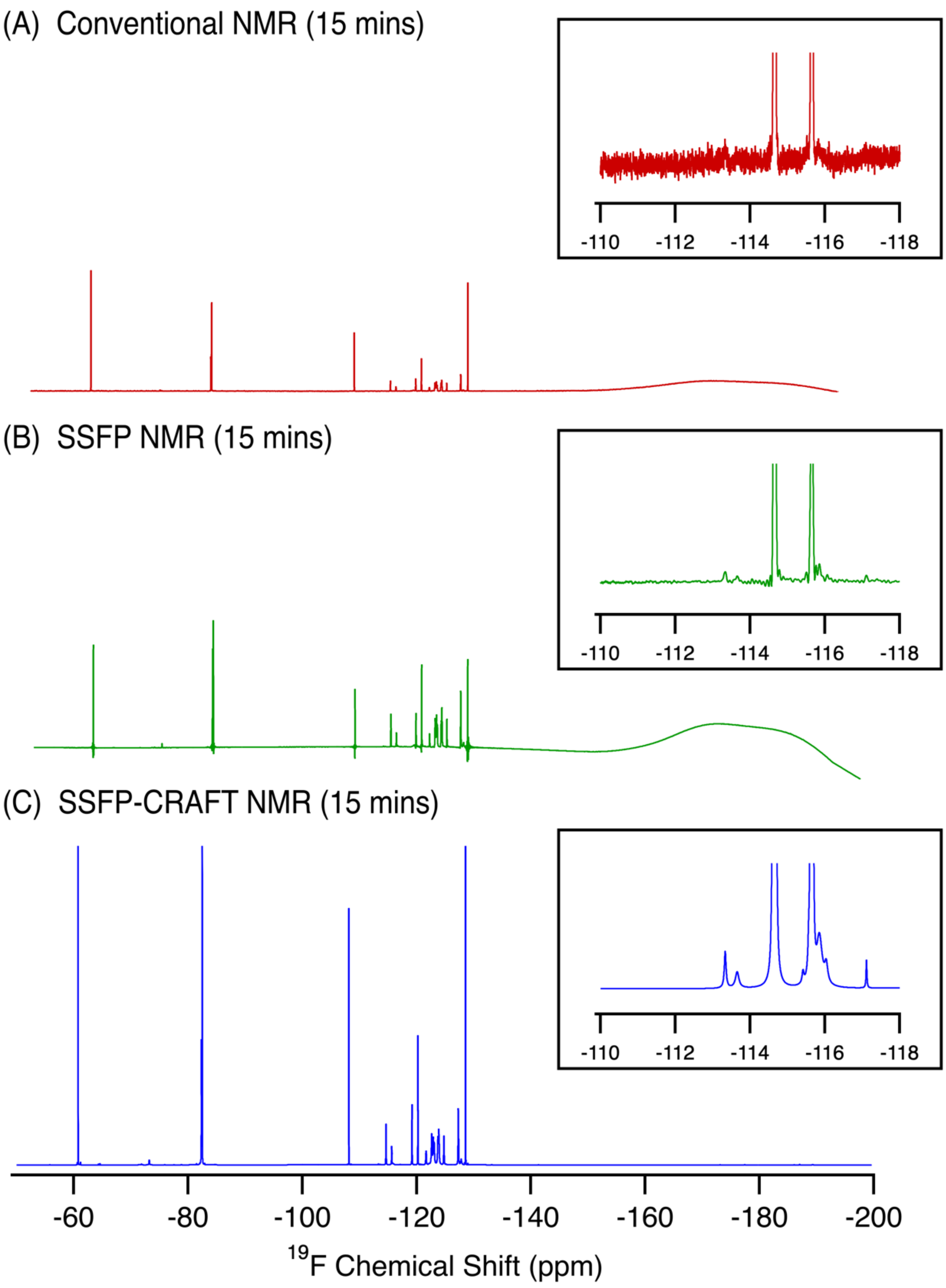

Figure S3: ^{19}F NMR of a mixture of known fluorinated compounds (15 min experiment time). A: Standard 1D ^{19}F NMR spectrum. B: SSFP ^{19}F NMR spectrum processed by Fourier transform. C: SSFP ^{19}F NMR spectrum processed by CRAFT. Vertical scaling is 1:1:1.

The results from the SSFP data processed using CRAFT instead of a Fourier transform highlight the advantage of the combined SSFP-CRAFT method. Figure 1 in the main manuscript shows that spin multiplicity is retained with the SSFP-CRAFT method which offers additional valuable information over SSFP alone. Notably, the CRAFT processing method suppresses artifacts, noise, and the probe background. This allows signals to be recovered which would normally be underneath the significant background which is the result of fluorine-containing glues and coatings used in the probe construction and exacerbated by the nature of the steady state approach. Figure S4 shows the region containing the probe background. Only using CRAFT and SSFP combined is it possible to identify the four resonances contained within this region. In Figure S4B there are indications that these resonances are present, however the signal to noise ratio is not sufficient for a confident identification. The CRAFT processing allows for confident determination of these resonances.

As noted in the main manuscript, CRAFT decimates the time-domain data into a tabular domain. A broad (and significant) probe background signal in the spectrum represents dominant, but fast decaying, signals in time domain perturbing several early points (as much as 500 to 600 microseconds – i.e., 50+ dwell time for typical ^{19}F spectral width of 100 kHz) in the FID. Had the acquisition been delayed by 500-600 microseconds the probe background signals would have decayed completely for all practical purposes and the FID would be dominated by the signals of interest. However, a Fourier transformation of such "delayed FID" will result in exceedingly large phase difference for signals across the spectrum (for example, adding as much as 18,000 to 21,600 degrees of linear phase error across a spectral width of 100 kHz) making it almost of impractical value. Analyzing such "delayed FID" in time-domain has no such practical constraint. As noted in the main manuscript, the CRAFT output is a table of frequency/amplitude/decay-rate/phase of every decaying sinusoid in the FID. For each such model, the amplitude and phase describe the magnitude and direction (in the rotating frame of reference), respectively, while the frequency and decay-rate describe the rate of change of phase and amplitude, respectively. Thus these 4 parameters are orthogonal to each other. Such computation from a "50-dwell delayed FID" would initially compute the amplitude and phase being that of the 51st data point but has no effect on the rate of change of amplitude and rate of change of phase. Thus, recomputing (and tabulating) the amplitude and phase of the 1st data point from those of the 51st data point is no more than solving

a basic exponential equation for each of the models. All CRAFT analysis in this study were performed after ignoring the first 50 complex points. The so-called “probe background” in CRAFT spectra is moot as those signals almost completely decayed in ~500 microseconds and do not exist in the “50-dwell delayed FID”. However, analyte peaks (slow decaying signals) that were hopelessly buried under the “probe background” are still very much present even after 500 microseconds and recognized by CRAFT. This is shown in Figure S4.

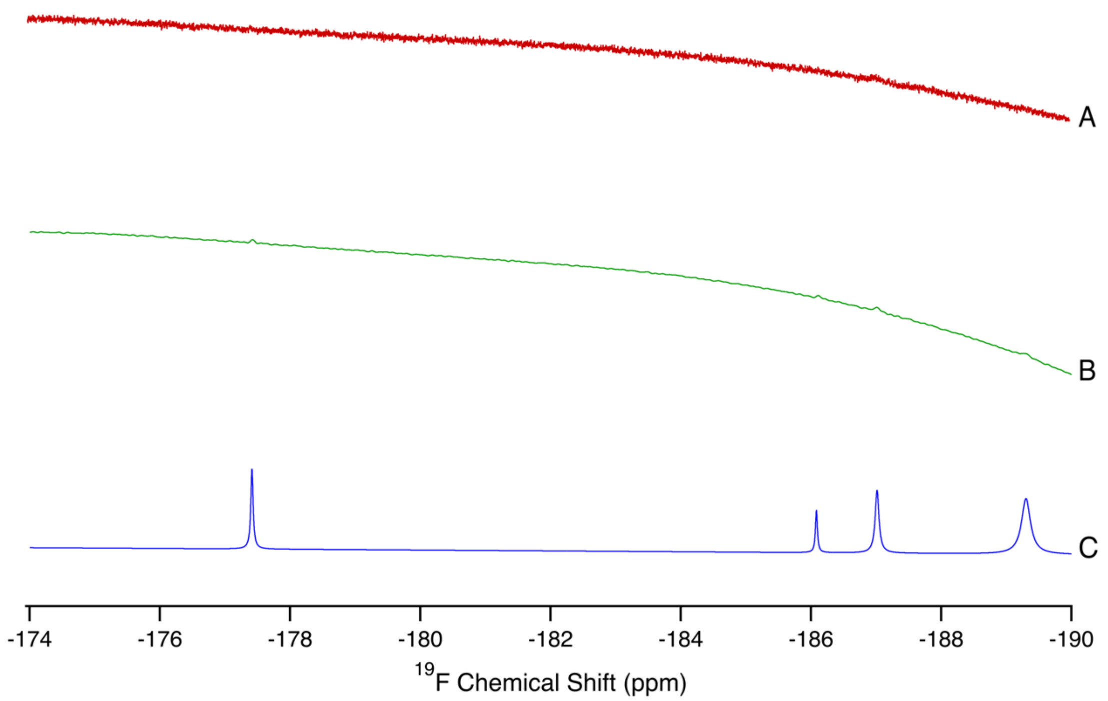

Figure S4: ^{19}F NMR spectrum of a mixture of known fluorinated compounds, focusing on the region where the probe background signal occurs. A: Standard 1D ^{19}F NMR spectrum. B: SSFP ^{19}F NMR spectrum processed by Fourier transform. C: SSFP ^{19}F NMR spectrum processed by CRAFT.

To demonstrate the SSFP-CRAFT results are truly representative of components present, Figure S5 shows a comparison between a 14-minute 1D NMR experiment (Figure S5A), a 14-minute SSFP and CRAFT NMR experiment of the same data (Figure S5B), and a 5 hour and 39-minute 1D NMR experiment (Figure S5C). It is clear from this data that in just 14 minutes of experiment time, SSFP + CRAFT generates a spectrum with near-identical line shape as the standard 5 hour and 39-minute 1D NMR experiment. The relative ratio of signals and the line shape is generally similar. This holds true for both the more intense resonances (Figure 2 in the manuscript) as well as near the level of the noise (Figure S5).

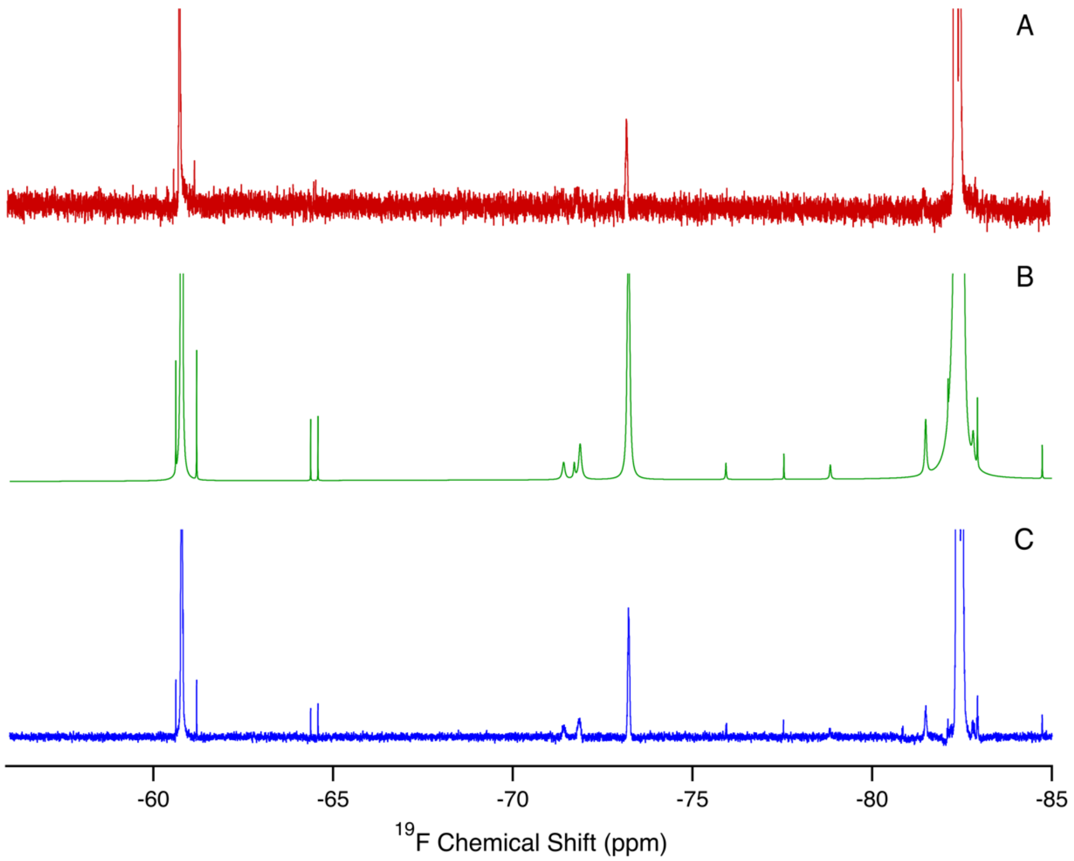

Figure S5: NMR spectra of a mixture of fluorinated compounds highlighting the similarity in relative intensity and line shape near the noise level between a 14-minute 1D ^{19}F NMR experiment (A), a 14-minute SSFP and CRAFT ^{19}F NMR experiment (B), and a 5-hour 1D ^{19}F NMR experiment (C).

1.4 Discussion of CRAFT model parameters

As CRAFT relies on probabilistic modelling, i.e. Bayesian analysis of the NMR data, few, if any parameters need to be adjusted in the fitting. However, for visualization a key parameter amplitude-to-noise ratio (A/N or amp2noise) is important. In practice, the detectability in the CRAFT method is threshold by the amplitude-to-noise ratio (A/N), rather by signal(height)-to-noise ratio. As a definition, A/N of 1 represents a sinusoid of amplitude A (LWHH = +/- 1 digital point) that would equate to the 1*RMS noise value. Considering noise is random and hence would be a normal (gaussian distribution) the A/N can be adjusted to investigate the confidence levels at which a potential peak rises above the threshold of the randomness of the noise. A/N above 3.3 is ~100% confidence, A/N of 2.5 is 99.7% confidence, etc. Figure S6 shows the result on the CRAFT spectrum as the A/N is varied and compares this result to a standard 1D NMR experiment. As the confidence of the model decreases, a handful of new resonances appear. Notably, this effect works in both "directions" as at A/N of 5, resonances are missing from the spectrum, a result of the model taking an overly cautious approach to data analysis. This raises an important question as to where to set this parameter for complex samples with unknown peaks. For this study, this was determined by repeating the approach in Figure S6 for the real-world samples. Namely, running a shorter SSFP and a conventional NMR for a longer period of time and seeing what A/N setting produced the most representative data. For all samples (except one) a setting of A/N of 2.5, or 99.7% confidence was found to be ideal and therefore used throughout this work. This would be our general recommendation for all unknown biological/environmental samples moving forward. The one exception was the spectra for the tap water sample which is discussed separately in the next section.

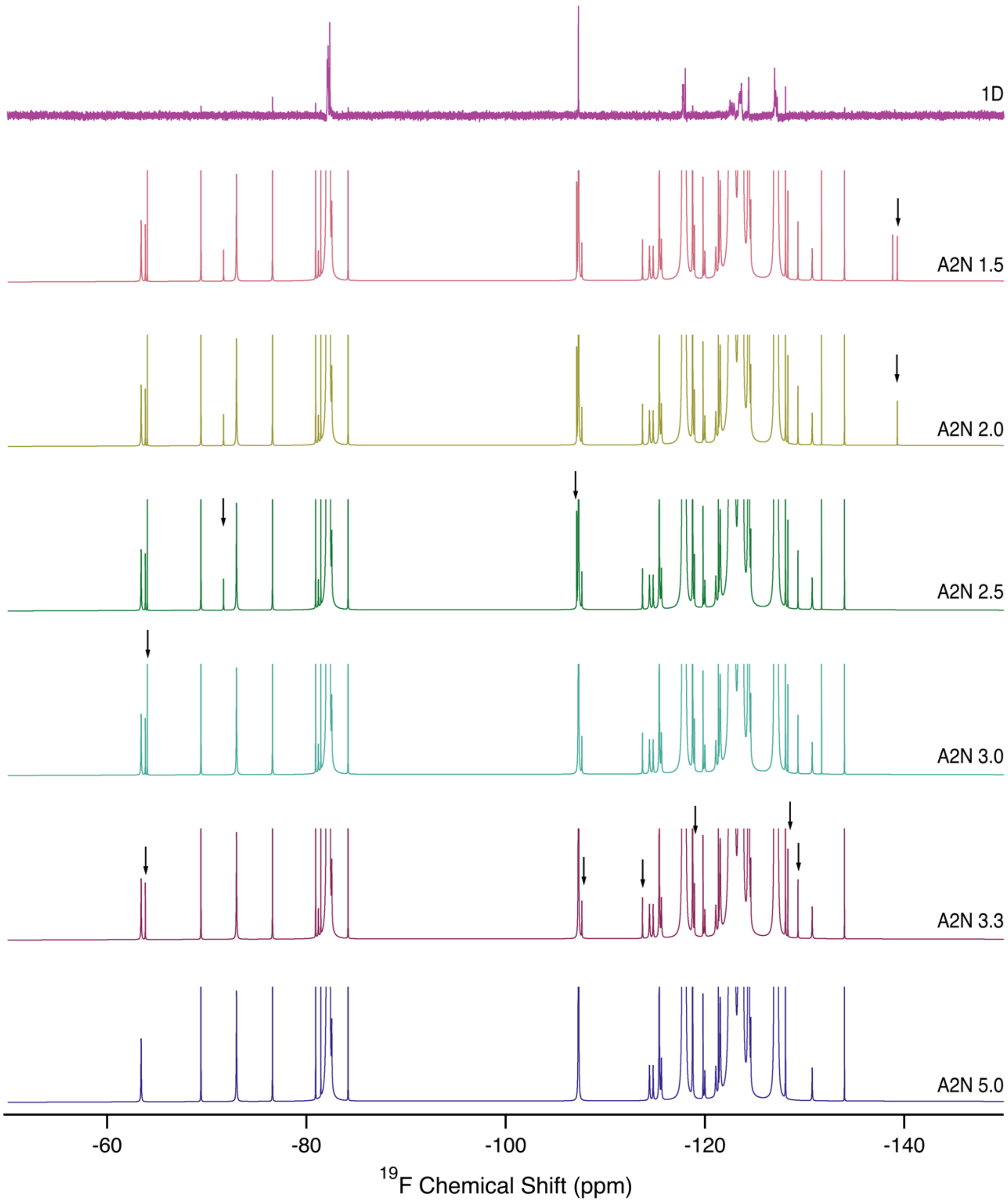

Figure S6: ^{19}F SSFP-CRAFT NMR spectra of the Lake Niapenco sample at varying amp2noise thresholds. Arrows indicate the appearance of new peaks as the threshold for visualization "A/N" is decreased.

The exception to the use of an A/N of 2.5 is the drinking water sample, which has exceedingly low concentrations of some compounds. In this case the signals themselves are so close to the noise level, that A/N is slightly too conservative and a setting of 2.5 means some critical peaks are missed. Figure S7 shows a comparison between the SSFP result, and CRAFT processing with A/N values of 1.5 and 2.0. In this instance, the SSFP helps identify which peaks are real and which are not, while CRAFT can help pull out these low intensity peaks, and those peaks which are twisted in phase. Between -55 and -90 ppm, we observe excellent agreement between the SSFP spectrum and the A/N 1.5 spectrum, while the A/N 2.0 spectrum is missing several resonances. All peaks identified by the CRAFT spectrum at 1.5 A/N have signatures in the SSFP data. Due to the low SNR in the SSFP data, the use of A/N 2.0 would miss these weaker peaks. Between -110 and -150 ppm the SSFP peaks are even weaker. All peaks present in the CRAFT spectrum have visible signatures in the SSFP data, except for the resonance at -144 ppm. In this instance, the ideal statistical threshold for the CRAFT processing may fall between A/N of 1.5 and 2.0. In such a scenario, where a low A/N setting suggests the presence a new peak, that may be of scientific significance, it would be best confirmed by running the SFFP experiment for longer and redoing the CRAFT analysis. As such, if used cautiously, lowering the A/N threshold could be a useful tool in tentatively identifying the presence of a critical peaks at the noise level, which would then need to be confirmed by additional concentration or more scans. Notably, the tap water was the only sample where the use of A/N had to be adjusted. For the remainder of samples, it was trivial to set the A/N at 2.5, or >99.7% confidence. This in large part means the CRAFT analysis was performed without any additional input from the user.

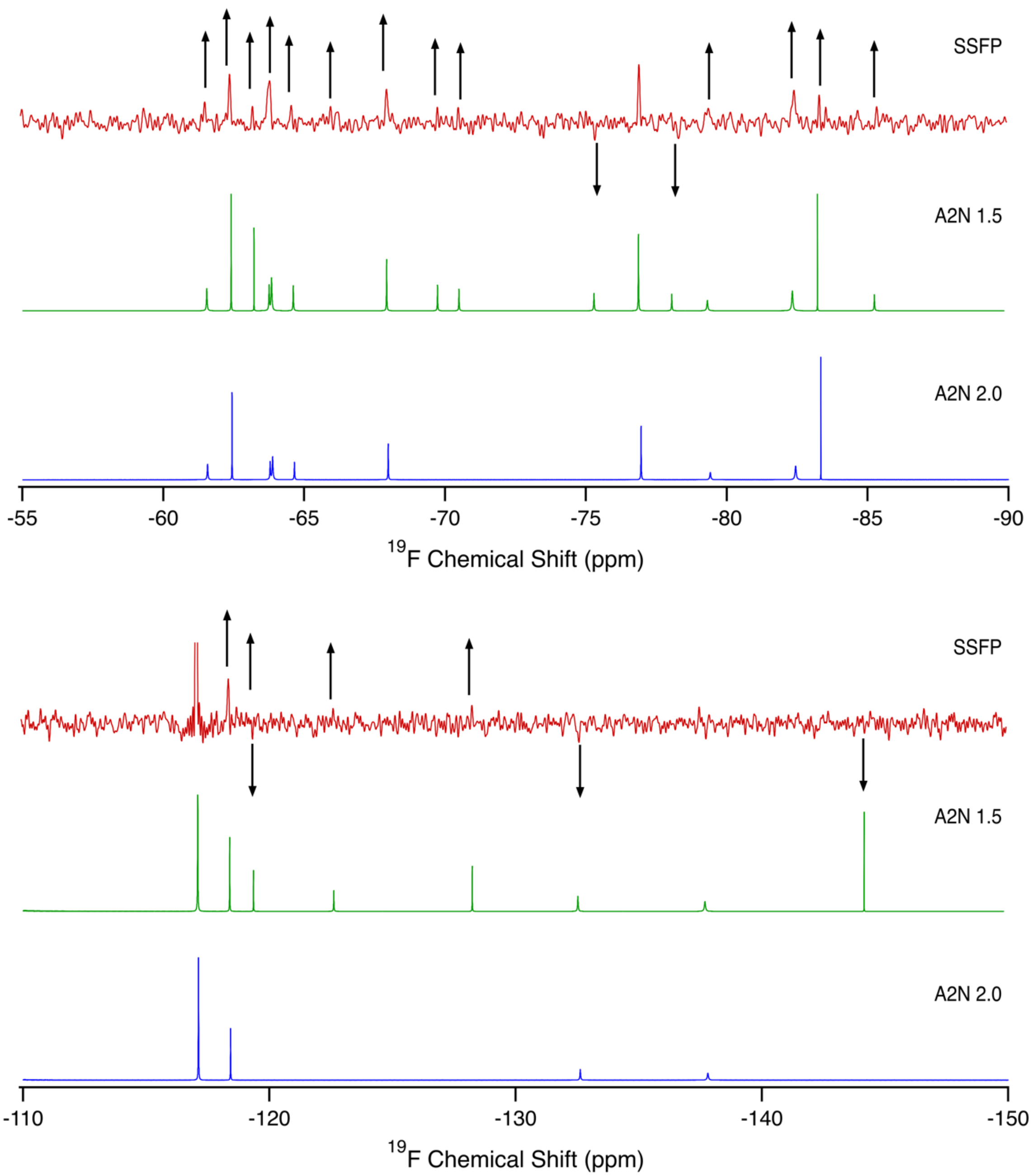

Figure S7: ^{19}F SSFP-CRAFT NMR spectra for the tap water sample showing the comparison between SSFP spectra, and processing with CRAFT at amp2noise (A/N) thresholds of 1.5 and 2.0. Arrows indicate discrete signals in the Fourier transform of the SSFP peak data can be identified albeit in some cases are twisted or inverted. The CRAFT processing method removes the artifacts and a setting of 1.5 A/N results in the best representation of the SSFP peaks in the CRAFT result. As this drinking water sample has a plethora of peaks near the noise level the lower threshold of 1.5 A/N is needed.

1.5 Limits of detection in complex samples

To provide an estimate of limits of detection for this method, we spiked PFOA into human whole blood at three concentrations: 1 µM, 10 µM, and 50 µM. Results are shown in Figure S8. PFOA in whole blood is a challenging sample, as PFOA can bind to human serum albumin. This creates line broadening, which can compromise detection limits versus PFOA in a solvent-based matrix. At 50 µM in 15 hours, standard NMR can just detect the analyte at ~3:1 SNR (i.e. at the limit of detection). On the other hand, even at 1 µM spiked concentration, SSFP-CRAFT is still able to recover signal from the main CF_2 chain. The results in an impressive ~50 fold increase in detection limit for PFOA in human blood.

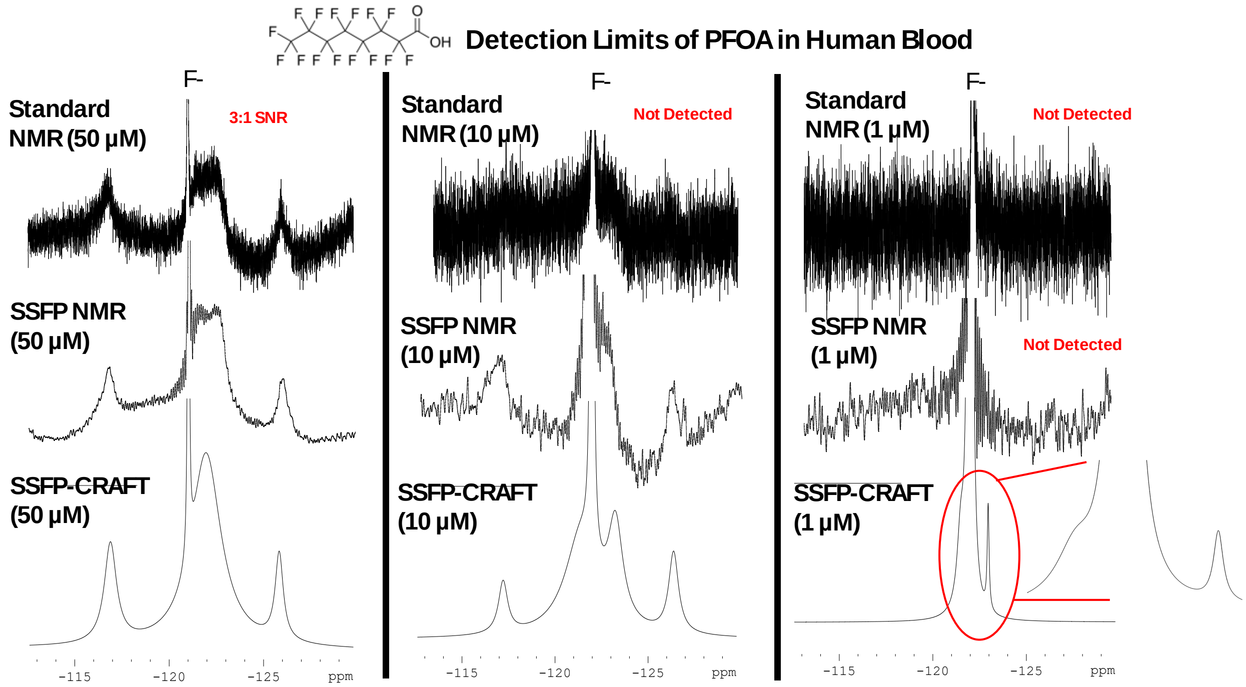

Figure S8: ^{19}F SSFP, ^{19}F standard 1D, and ^{19}F CRAFT + SSFP NMR of PFOA spiked in human whole blood at 1 µM, 10 µM and 50 µM concentrations. All experiments were run for a total of 15 h experiment time. At 1 µM SSFP-CRAFT is able to detect the larger $(CF_2)_n$ main chain resonances while smaller peaks are below detection limit. The large peak at ~121ppm is naturally occurring fluoride in blood and not from PFOA.

1.6 Method Limitations

Although some papers describe quantitative studies involving SSFP,[14] especially using time-domain NMR spectroscopy, we would not recommend this as a fully quantitative approach for real world environmental and biological matrices where the compounds present are unknown and vary greatly from polymers to small molecules without careful consideration of experimental parameters. This is because the efficiency of SSFP will vary greatly under the same experimental conditions. Slower relaxing molecules require longer acquisition times to achieve a return of the same equilibrium magnetization that is observed by more quickly relaxing molecules. While SSFP could, in more focused studies, be applied in a quantitative manner if T_1/T_2 effects are measured and fully accounted for,[14] or via spiking (if standards are available), in general we suggest SSFP should be primarily used as a rapid complementary screening tool to show what is truly present in a sample, to guide subsequent NMR or MS based analyses. In addition, it could have considerable application for monitoring processes such as bioaccumulation or photodegradation, where relative quantification across a sample series can be used to monitor the change of species over time.

The best solution for quantification will be to explore the optimal use of relaxation agents in combination with SSFP-CRAFT. The ideal situation being where the difference in relaxation of analytes in a mixture is normalized by the relaxation agent, and any spectral broadening caused by the agents is less than (or on par with) the spectral broadening from SSFP. The natural lineshape could then be fully recovered by the CRAFT analysis. We have explored this avenue briefly in section 1.7, although additional optimizations could further improve these initial results.

Another limitation is that current 2D NMR experiments such as a ^{19}F-^{19}F COSY and ^{19}F-^{13}C HSQC which could be useful for identification in overlapping regions, are not easily combined with SSFP NMR. This is because accurate flip angles (required to generate the correct state for coherence selection), and evolution delays (required to form the second dimension) will lead to the loss of the steady-state magnetization. We would suggest however, that should the 1D SSFP-CRAFT result produce a resonance of interest, it could provide the evidence needed to warrant a (potentially) lengthy 2D acquisition at low concentration, or bulk concentration of the sample to obtain 2D NMR data providing unique insight. Techniques including ^{1}H-^{19}F COSY, ^{19}F-^{19}F COSY and ^{19}F-^{13}C HSQC would be useful here.

1.7 Investigation of the potential for SSFP to perform quantitative measurements

To examine the quantitative potential of the combined SSFP-CRAFT approach under various conditions we ran a standard PFAS mixture containing six unique fluorinated compounds: 3,5-bistrifluoromethyl benzoic acid (3,5B-TFMBA), trifluoroacetic acid (TFA), perfluorooctanoic acid (PFOA), perfluoropropionic acid (PFPrA), 6:2 fluorotelomer alcohol (6:2 FTOH), and Methyl-2,2,3,3-tetrafluoropropyl ether (MeF-Ether). Each of these compounds has a distinct, non-overlapping resonance in the ^{19}F NMR spectrum to allow for accurate peak integration. A known concentration of each compound was spiked into deuterated methanol containing either 0 mg/mL, 4 mg/mL, or 8 mg/mL of paramagnetic relaxation agent chromium acetylacetonate (Cr) and internal standard 4,4'-difluorobenzophenone (4,4'-DFB). ^{19}F NMR spectra were acquired either under quantitative 1D conditions (D1 of 30s, more than 5x the longest T1), or using the combined SSFP-CRAFT approach, with varied SSFP acquisition times (AQ) of 30, 50, 100, or 200 ms. The goal was to investigate the use of a relaxation agent to shorten T1 times, as well as the influence of the FID acquisition time (AQ) on the quantification. Results were also compared against spike and recovery experiments performed using an optimized liquid chromatography tandem mass spectrometry method. Results are shown in Table S2, S3, and Figure S9.

Table S2: Investigation of the quantitative capabilities of the SSFP-CRAFT approach. Table provides compound-specific percent recoveries for a spike and recovery experiment. All values were measured in triplicate and error represents the 95% confidence interval.

	**Compound**	**Quantitative 1D NMR**	**SSFP 30ms AQ**	**SSFP 50ms AQ**	**SSFP 100ms AQ**	**SSFP 200ms AQ**	**LC-MS/MS**
0 mg/mL Cr	3,5B-TFMBA	103.8 ± 4.1	204.2 ± 46	172.0 ± 17	152.2 ± 36	104.1 ± 45	92.8 ± 7.9
	TFA	113.9 ± 2.3	193.4 ± 59	80.5 ± 14	61.2 ± 12	119.6 ± 12	56.7 ± 35
	PFOA	103.1 ± 6.1	87.9 ± 20	97.8 ± 18	110.4 ± 31	78.1 ± 2.5	100.6 ± 2.1
	PFPrA	106.4 ± 2.6	138.6 ± 68	172.3 ± 23	93.8 ± 76	51.3 ± 9.9	96.5 ± 11
	6:2 FTOH	98.2 ± 5.1	164.1 ± 52	199.9 ± 12	175.6 ± 47	146.4 ± 4.7	23.2 ± 31
	MeF-Ether	99.6 ± 3.2	154.2 ± 66	134.4 ± 11	84.9 ± 20	50.7 ± 21	Not detected
4 mg/mL Cr	3,5B-TFMBA	100.8 ± 11	104.9 ± 21	95.7 ± 5.3	85.0 ± 7.1	97.3 ± 10	
	TFA	97.5 ± 12	78.9 ± 13	68.5 ± 2.2	68.1 ± 2.2	83.9 ± 9.8	
	PFOA	105.7 ± 16	77.4 ± 11	94.6 ± 4.6	100.1 ± 10	98.7 ± 19	
	PFPrA	89.1 ± 1.9	94.5 ± 18	79.4 ± 7.2	83.7 ± 7.7	85.6 ± 8.6	
	6:2 FTOH	97.9 ± 2.3	94.5 ± 8.4	95.2 ± 3.6	96.4 ± 5.9	95.6 ± 7.8	
	MeF-Ether	97.2 ± 7.8	71.5 ± 7.1	77.8 ± 1.8	79.7 ± 5.3	87.6 ± 14	
8 mg/mL Cr	3,5B-TFMBA	98.7 ± 7.3	88.8 ± 1.7	101.1 ± 4	101.7 ± 0.3	95.4 ± 6.2	
	TFA	100.5 ± 5.2	75.3 ± 5.9	81.5 ± 0.5	83.9 ± 0.2	102.1 ± 5.3	
	PFOA	105.3 ± 2.5	95.8 ± 22	94.5 ± 4.2	100.7 ± 1.9	106.2 ± 12	
	PFPrA	96.9 ± 2.3	75.7 ± 1.9	84.5 ± 2.6	86.2 ± 1.8	88.2 ± 14	
	6:2 FTOH	101.0 ± 1.7	79.7 ± 2.2	91.0 ± 0.7	92.0 ± 4.3	100.3 ± 6.5	
	MeF-Ether	98.3 ± 4.3	69.2 ± 4.7	79.4 ± 7.3	72.5 ± 12	89.2 ± 7.1	

Table S3: Averaged percent recovery values for all analytes determined using ^{19}F NMR, error represents the 95% confidence interval

	**1D NMR**	**SSFP (30ms aq)**	**SSFP (50ms aq)**	**SSFP (100ms aq)**	**SSFP (200ms aq)**
0 mg/mL Cr	104.2 ± 10	148.9 ± 32	136.8 ± 34	111.2 ± 29	92.9 ± 26
4 mg/mL Cr	98.0 ± 6.4	88.8 ± 9.5	87.3 ± 8.8	87.6 ± 8.8	92.7 ± 4.9
8 mg/mL Cr	100.1 ± 7.7	83.5 ± 8.5	90.3 ± 6.4	92.7 ± 4.9	97.3 ± 4.9

It is clear from results in Table S3 that there is a trending improvement in accuracy of the NMR method as the concentration of paramagnetic relaxation agent increases as well as the length of acquisition time in the SSFP increases. As expected, faster relaxing compounds such as larger PFAS demonstrated less variability in their absolute integrals than longer relaxing compounds and tend to be overestimated in concentration given their excellent detection. As the acquisition time becomes longer and the T1 time shortened this discrepancy decreases as nuclei become more equal, in terms of their total decay captured by the FID. Currently the most optimal SSFP conditions in this proof-of-concept test were AQ=200ms with 8mg/mL Cr, which were on average, within 3% of the true value with the largest deviation still less than 10%. However, despite these improvements in accuracy with the inclusion of paramagnetic relaxation agent and increasing the acquisition time it is important to note that the optimal quantitative NMR approach remains the conventional 1D NMR experiment, which demonstrated high accuracy and precision for the measurement of fluorinated compounds in this example. Further optimization of the SSFP-CRAFT method in terms of type and amount of relaxation agent should bring the SSFP-CRAFT results even closer to those in standard 1D quantitative NMR.

Results from this experiment were additionally compared against LC-MS/MS analysis. This demonstrated that the accuracy and precision was comparable to conventional 1D NMR only for those compounds which are amenable to electrospray ionization mass spectrometry. The fluorinated ether acid was not detected by mass spectrometry, while 6:2 FTOH suffered from poor ionization, resulting in average percent recovery values of 23.2%. Trifluoroacetic acid also suffered in mass spectrometry owing to its poor retention on conventional reversed phase chromatography columns combined with only one multiple reaction monitoring transition, leading to poor identification of TFA from the solvent front. This helps to highlight the advantage NMR has in detecting compounds which may not be amenable to certain mass spectrometry techniques.

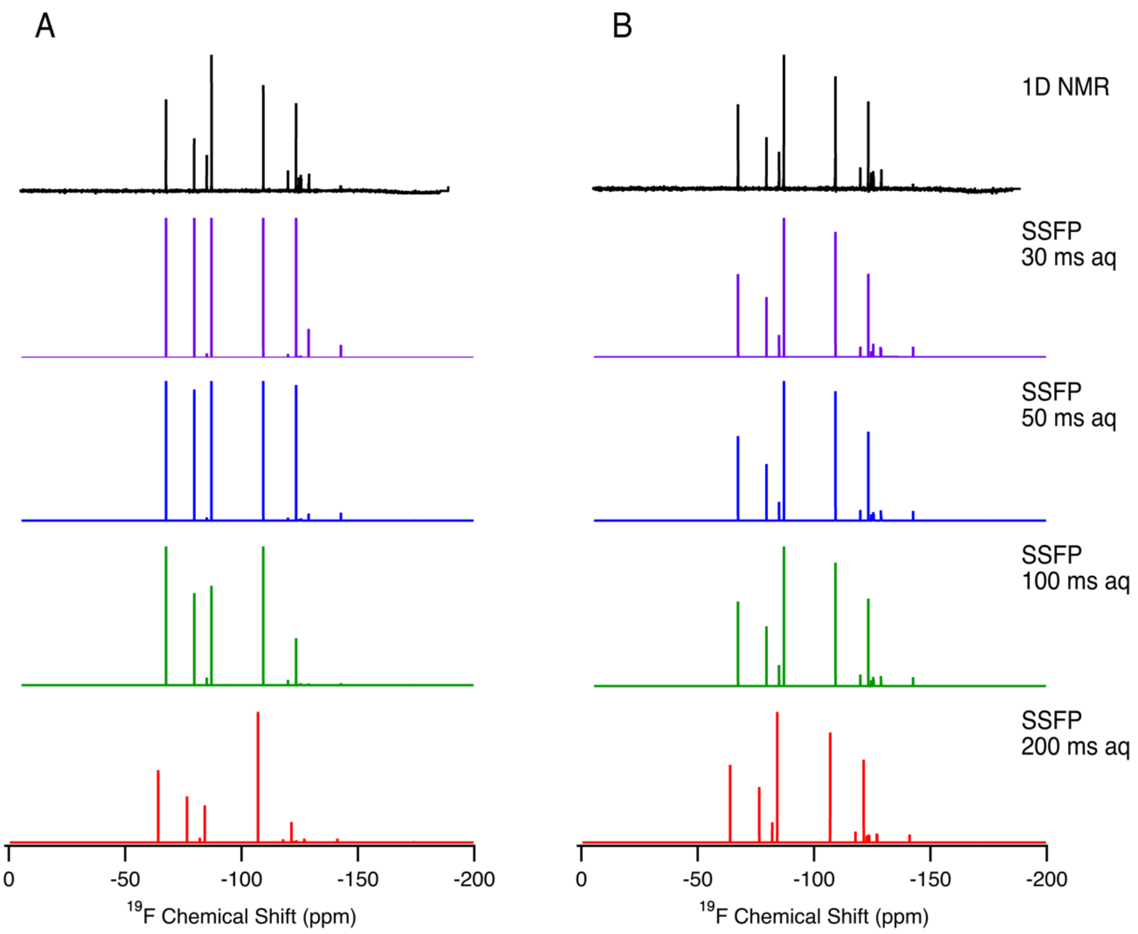

Figure S9: Comparison of conventional ^{19}F 1D NMR and SSFP-CRAFT spectrum for a mixture of known fluorinated compounds to examine the quantitative potential of the technique. A: No paramagnetic relaxation agent used, B: inclusion of 8 mg/mL chromium acetylacetonate. As the acquisition time (AQ) in the SSFP method increases and with the inclusion of relaxation agent it is apparent the relative ratios of signals are better retained, at the cost of overall experiment time.

Section 2 – Experimental

2.1 Sample preparation

Chemical and Materials

NMR solvents D_2O and MeOD-d_4, internal standards 4,4'-difluorobenzophenone (99.9%, δ -108 ppm) and 4,4,4-trifluoro-1-butanol (99%, δ -63 ppm), and extraction solvent methanol were purchased from Millipore Sigma. Solid-phase extraction (SPE) cartridges for sample extraction were purchased from Waters as Oasis weak anion exchange (WAX) and hydrophilic-lipophilic balanced (HLB) sorbents with 500 mg sorbent mass.

Drinking water

Drinking water was taken directly from municipal water sources. Drinking water (4-litres) was passed through two solid-phase extraction (SPE) cartridges in series: first a WAX cartridge to capture strongly acidic PFAS, followed by an HLB cartridge to capture remaining fluorine-containing compounds. The 500 mg sorbent mass of each cartridge is sufficient for the extraction of up to at least 4-litres of water before mass loading becomes a concern.[13,22] The extraction was run according to the manufacturer's directions for each sorbent type, and the extracts from each cartridge combined, evaporated under a gentle stream of nitrogen at 20 °C, and reconstituted in MeOD-d_4 containing internal standard 4,4'-difluorobenzophenone (0.5 mM, δ -108 ppm) for NMR analysis.

House paint

Exterior house paint from a major international brand was accurately weighed (100.1 mg) prior to dilution with 10% D_2O and internal standard 4,4,4-trifluoro-1-butanol (0.5 mM, δ -63 ppm). The sample was centrifuged at 5000 rpm for 5 minutes to removed suspended particulate prior to analysis by NMR.

AFFF-impacted surface waters

The preparation of the surface water sample, Lake Niapenco, is previously described.[22] In short, 120 L of surface water was collected, aliquot into 4-litre fractions, and extracted through both WAX and HLB SPE cartridges in series. Extracts were reconstituted in MeOD-d_4 containing 4 mg/mL chromium (III) acetylacetonate and 0.5 mM internal standard 4,4'-difluorobenzophenone (0.5 mM, δ -108 ppm).

Polar bear liver

Archived polar bear liver (100.4 mg) from the Southern Hudson Bay population were extracted by homogenization with deuterated methanol containing internal standard 4,4'-difluorobenzophenone (0.5 mM, δ -108 ppm). The homogenized sample was transferred directly to an NMR tube for analysis. These samples were donated from the Canadian National Wildlife Specimen Bank as described previously.[36] These samples were collected by the Canadian government in collaboration with indigenous sustenance hunters and were not sampled for this study.

Biosolids

Pelletized biosolids were obtained from Nutripel (Ontario, Canada), who collected and processed wastewater treatment plant sludge from the Ashbridges Bay wastewater treatment plant in Toronto, Canada. The pellets (35.02 g) were sonicated three times in methanol for 1 hour. The methanol extracts were combined, evaporated under a gentle stream of nitrogen at 20 °C, and reconstituted in MeOD-d_4 containing internal standard 4,4'-difluorobenzophenone (0.5 mM, δ -108 ppm).

Human serum

Human serum was purchased from BioIVT as a pooled sample of 1000 participants (BioIVT HUMANSRM-0000351). No human serum was collected for this study. The serum (1.0104 g) was vortexed three times in MeOD-d_4 (2 mL) followed by centrifugation. The combined extracts were evaporated under a gentle stream of nitrogen at 20 °C, and reconstituted in MeOD-d_4 containing internal standard 4,4'-difluorobenzophenone (0.5 mM, δ -108 ppm).

Human Blood Sample spiked with PFOA

The human blood sample spiked with PFOA to determine detection limits was provided by an anonymous donor and did not involve recruitment or enrollment of human subjects. The University of Toronto ethics officer confirmed additional ethics approval was not required for this study. PFOA at concentrations of 1 µM, 10 µM, and 50 µM were directly spiked into the whole blood sample using D_2O as a carrier solvent and as a spectrometer lock. No further sample extraction took place prior to analysis by ^{19}F NMR. The ratio of human blood to D_2O was consistent between each concentration at 10% D_2O (v/v). For investigation of detection limits, the alkyl-CF_2 resonance was selected over the alkyl-CF_3 resonance as the overlap of alkyl-CF_2 signals lead to a higher signal intensity resultant from 10 fluorine nuclei with similar chemical shifts. Although this resonance was broader than that of the terminal alkyl-CF_3, lineshape was recovered using the combined SSFP-CRAFT method.

Method blanks

A method blank was designed to encompass all extraction methods used in this study. First, 8-litres of MilliQ (18 moHm resistance) water were extracted in series by WAX and HLB SPE cartridges. Alongside this, 20 g of laboratory sand (Millipore Sigma) that had been previously washed with methanol was extracted 3 times by methanol washes of 30 mL. Finally, washed lab sand was combined with deuterated methanol (2 g sand, 2 mL d4-MeOD), vortexed, sonicated, and centrifuged. All methanol extracts were evaporated to near-dryness using a nitrogen evaporator at 22 °C. The extracts were combined and reconstituted in deuterated methanol alongside internal standard 4,4'-difluorobenzophenone (0.5 mM final concentration).

This blank sample was acquired using ^{19}F SSFP NMR for 18 hours (see Figure S10). After this acquisition, three resonances were visible in addition to the internal standard at -108 ppm. A resonance at -64 ppm which corresponding to aromatic CF_3 groups, a resonance at -77 ppm, corresponding to trifluoroacetic acid, and a resonance at -120 ppm, corresponding to fluoride. All spectra collected as part of this study were compared against the blank experiment, and in any case where resonances were overlapping, the blank had notably lower signal intensity and peak area than any experimental sample.

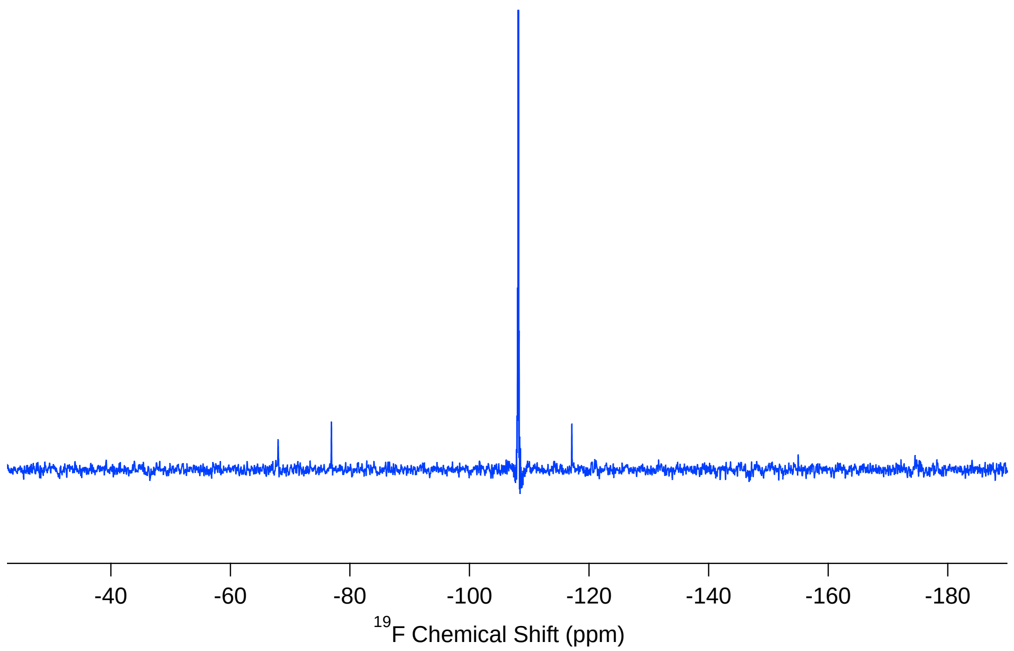

Figure S10: ^{19}F SSFP NMR of the combined method blank sample, described above, showing two resonances present in addition to the internal standard at -108 ppm and inorganic fluorine at -118 ppm. Total scans: 3,276,800, with experiment time of 18 hours.

2.2 NMR Experimental parameters

NMR experiments were performed on a Bruker Avance III NMR spectrometer equipped with a $^{19}F/^{1}H$ Selective (SEF) probe, operating at 11.7 T. The spectrometer was tuned to observe frequencies for ^{1}H and ^{19}F at 500.28 and 470.73 Hz, respectively. All spectra were acquired at room temperature (298 K).

1D NMR spectra were acquired as $^{19}F\{^{1}H\}$ using ^{1}H waltz-16 decoupling during acquisition. 320,000 time domain (TD) points were collected for each scan, with a frequency offset (O_1P) of -100 ppm, acquisition time of 1.70 s, spectral width (SW) of 200 ppm, and recycle delay (D1) of 5.0 s. Number of scans ranged between 128 to 6144 scans.

^{19}F SSFP spectra were collected using the same experimental parameters for decoupling, O_1P, and SW as the 1D experiment. A 4-step phase alternation was applied, as previously described.[14] In short, the phase alternation allows a uniform response across the entire spectral region in SSFP while overcoming the limitation of "sideband-like" nulls that can arise otherwise. See Figure 2c in Moraes et al. for additional information.[14] The optimal flip angle was determined empirically for each sample to obtain maximum signal per unit.

In general, a pulse angle between 45° and 75° was used (see Table S1). An acquisition time of 29 ms with D1 of 1 ms was used. The 1D ^{19}F-SSFP-NMR spectra were collected using 5614 TD points. Numbers of scans ranged between 28172 and 2.6 million. The pulse sequence code for implementation on a Bruker spectrometer is provided in supporting section S3.

2.3 Data processing

Processing of conventional 1D ^{19}F NMR spectra

Data was processed in Topspin 4.3.0. 1D ^{19}F-NMR spectra were processed using an exponential function corresponding to 1 Hz line broadening (LB) in the transformed spectrum and automatic baseline correction.

Processing of 1D SSFP ^{19}F NMR spectra by Fourier transform

First backwards complex linear prediction was applied to remove some of the very broad probe (>20KHz) background. As SSFP emphasizes components with fast relaxation, components used in the probe construction (glues, Teflon, outside the coil) become visible during the very high number of scans. Next a Trapezoid function is applied which forces the truncated FID to taper to zero to help reduce truncation artifacts. An exponential function is then matched to the decay envelope of the FID. Finally, additional forward linear prediction is applied that helps extend the length of the FID, which in turn improves resolution and further eliminates truncation artifacts (i.e. “wiggles” superimposed onto the spectrum). The number of coefficients and number of points in each prediction was optimized on a per-sample basis to obtain the maximum SNR and to minimize spectral artifacts. SNR was calculated in Topspin 4.3.0 using a signal and noise region which was defined for each sample, but always consistent between 1D and SSFP spectra. A macro for implementing this processing automatically in Topspin is provided in subsequent sections.

Processing of 1D SSFP ^{19}F NMR spectra by CRAFT

In CRAFT processing the raw input FID is subjected to Bayesian decimation with no pre-processing requirements. The typical CRAFT workflow and detailed technical aspects are described in references 20 and 21 in the manuscript. All the tools needed to build CRAFT workflow is available in some form or another in all commercial NMR software and/or available as free downloads. The CRAFT package used in this study is built on OpenVnmrJ software (open

source) platform and is commercially available. The input FID is converted from their native vendor format to OpenVnmrJ format and subjected to CRAFT processing. There are two unique aspects of the input data used in this study. (i) Irrespective of conventional data or SSFP data, they have probe background signal; and (ii) the SSFP data are (by their very nature) truncated, typically between 30 or 50 ms acquisition time. CRAFT decimation is done after skipping the first 50 complex time-domain points. This translates as equivalent to ~500 microsecond delayed acquisition. Unlike Fourier transform processing, CRAFT processing requires no pre-processing (aka apodization). The raw input FIDs are subjected to decimation as they are. The CRAFT results are tabular representations of individual models that adds up to the input FID. They are visually examined and/or compared with FT results (as in various figures in the manuscript), by first simulating a FID followed by conventional FT. However, CRAFT simulations (the length of the simulated FID in particular) are not constrained by the input acquisition time, and hence need not be truncated. In this study all CRAFT simulations are extended to 1.2-1.3 sec, i.e., equivalent to ~250,000 complex time domain points for a 100 kHz spectral width. Thus, the linewidth of the resonances in CRAFT spectra are minimally perturbed by any significant apodization, nor do they suffer from typical truncation wiggles.

Section 3 – Pulse Code and Processing Scripts

3.1 ^{19}F SSFP Pulse program code

```
"d11=30m"
"DELTA=d1-10m"
"acqt0=-p1*0.66/3.1416"
1  ze
  d11 pl12:f2
2  30m do:f2
  10u pl12:f2
  100m cpd2:f2
3  (p1 pl1 ph1^):f1
   d3
   lo to 3 times l0
4  (p1 pl1 ph1^):f1
   gosc ph11
   d1 ipp11
   lo to 4 times l1
5  (p1 pl1 ph2^):f1
   d3
   lo to 5 times l0
6  (p1 pl1 ph2^):f1
   gosc ph12
   d1 ipp12
   lo to 6 times l1
7  (p1 pl1 ph3^):f1
   d3
   lo to 7 times l0
8  (p1 pl1 ph3^):f1
   gosc ph13
   d1 ipp13
   lo to 8 times l1
9  (p1 pl1 ph4^):f1
   d3
   lo to 9 times l0
10  (p1 pl1 ph4^):f1
   gosc ph14
   d1 ipp14
   lo to 10 times l1
  30m do:f2 mc #0 to 2 F0(zd)
exit

ph1=  0
ph11= 0
```

ph2= 0 1 2 3
ph12= 0 1 2 3

ph3= 0 2
ph13= 0 2

ph4=   0 3 2 1
ph14= 0 3 2 1

Description:
;pl1 : f1 channel - power level for pulse (default)
;pl12: f2 channel - power level for CPD/BB decoupling
;pl13: f2 channel - power level for second CPD/BB decoupling
;p1 : f1 channel - 90 degree high power pulse (optimize for max SNR)
;d1 : relaxation delay; normally short (1ms), optimize Tp using AQ time
;d3 : delay for purge block
;l0 : loop for purge block
;l1 : scans per block (total scans = 4 *l1)
;d11: delay for disk I/O   [30 msec]
;ns: 1
;cpd2: decoupling according to sequence defined by cpdprg2
;pcpd2: f2 channel - 90 degree pulse for decoupling sequence

3.2 Macros for processing SSFP data in TopSpin by Fourier transform

Samples can be processed using backward linear prediction only or processed using a combination of backwards and forwards linear prediction. The combination of backwards and forwards prediction results in sharper peaks. A balance should be achieved by varying the prediction parameters to minimize baseline distortions resulting from truncation of the FID without introducing artifacts resulting from the processing. Combining backwards and forwards linear prediction is not inherently straightforward to set up in TopSpin. As a result, we provide the following set of commands for data processing. SSFP data must first converted from digitally filtered to analog data using the *convdta* command in TopSpin. The following commands are executed on the analog data and are best converted to a macro for simplicity. The macro will ask for the dataset number where the analogue data to stored, and asked for ask the line broadening to be used, all other parameters are set automatically.

convdta
LB
TM1 0
TM2 0
SI 32768
efp
WDW no
FT_mod no
PH_mod no
BC_mod no
ME_mod LPfc
NCOEF 2048
LPBIN 4096
TDoff 0
trf
FT_mod fqc
ME_mod LPbc
NCOEF 48
LPBIN 0
TDoff 48
PH_mod pk
em
tm
trfp
abs

The emphasized number of linear prediction coefficients (**NCOEF**) and number of points for linear prediction (**LPBIN**) parameters for the complex linear forward prediction (LPfc) are optimized on a per-sample basis. We suggest starting with NCOEF of 512 and LPBIN of 2048, and increasing by doubling each value until spectral artifacts appear. Users will also need to experiment with value used for exponential function (LB in Hz) which should be set such that the exponential decay matches that of the FID. In addition, users should adjust the *NCOEF* 48, *TDoff* 48, used in the second half of the macro which control the backwards linear prediction, used for the suppression of the very broad probe background resulting from solids used in its construction. Probe background can be particularly evident in ^{19}F NMR, as many glues and plastics related to probe construction are highly fluorinated. Note, section 3.2 does not apply if CRAFT is used to process the SSFP data, as CRAFT is applied to the raw FID direction and Fourier transform is completely avoided. Data processing for CRAFT is covered in section 2.3 of this supporting information.
